# Ecogenomics and Taxonomy of Cyanobacteria Phylum

**DOI:** 10.3389/fmicb.2017.02132

**Published:** 2017-11-14

**Authors:** Juline M. Walter, Felipe H. Coutinho, Bas E. Dutilh, Jean Swings, Fabiano L. Thompson, Cristiane C. Thompson

**Affiliations:** ^1^Laboratory of Microbiology, Institute of Biology, Federal University of Rio de Janeiro, Rio de Janeiro, Brazil; ^2^Radboud Institute for Molecular Life Sciences, Centre for Molecular and Biomolecular Informatics, Radboud University Medical Centre, Nijmegen, Netherlands; ^3^Theoretical Biology and Bioinformatics, Utrecht University, Utrecht, Netherlands; ^4^Laboratory of Microbiology, Ghent University, Ghent, Belgium; ^5^Center of Technology - CT2, SAGE-COPPE, Federal University of Rio de Janeiro, Rio de Janeiro, Brazil

**Keywords:** microbial ecology, ecological niches, charting biodiversity, genome-based microbial taxonomy, metagenome, high-throughput sequencing technology

## Abstract

Cyanobacteria are major contributors to global biogeochemical cycles. The genetic diversity among Cyanobacteria enables them to thrive across many habitats, although only a few studies have analyzed the association of phylogenomic clades to specific environmental niches. In this study, we adopted an ecogenomics strategy with the aim to delineate ecological niche preferences of Cyanobacteria and integrate them to the genomic taxonomy of these bacteria. First, an appropriate phylogenomic framework was established using a set of genomic taxonomy signatures (including a tree based on conserved gene sequences, genome-to-genome distance, and average amino acid identity) to analyse ninety-nine publicly available cyanobacterial genomes. Next, the relative abundances of these genomes were determined throughout diverse global marine and freshwater ecosystems, using metagenomic data sets. The whole-genome-based taxonomy of the ninety-nine genomes allowed us to identify 57 (of which 28 are new genera) and 87 (of which 32 are new species) different cyanobacterial genera and species, respectively. The ecogenomic analysis allowed the distinction of three major ecological groups of Cyanobacteria (named as i. Low Temperature; ii. Low Temperature Copiotroph; and iii. High Temperature Oligotroph) that were coherently linked to the genomic taxonomy. This work establishes a new taxonomic framework for Cyanobacteria in the light of genomic taxonomy and ecogenomic approaches.

## Introduction

Earth is home to nearly one trillion (10^12^) microbial species that have evolved over ~4 billion years (Locey and Lennon, 2016). Cyanobacteria emerged ~3 billion years ago, ushering Earth's transition from anoxygenic to oxygenic conditions through photosynthesis (Schirrmeister et al., 2011a). Throughout their evolution, Cyanobacteria became one of the most diverse and widely distributed Prokaryotes, occupying many niches within terrestrial, planktonic, and benthic habitats. Their long history evolved in a broad heterogeneity comprising unicellular and multicellular, photosynthetic and non-photosynthetic (i.e., Melainabacteria) (Schirrmeister et al., 2011a; Di Rienzi et al., 2013; Soo et al., 2014), free-living, symbiotic, toxic and predatory organisms (Soo et al., 2015), with genomes sizes ranging from 1 to 10 Mb (Shih et al., 2013). Here we consider Cyanobacteria phylum as consisting only of oxygenic phototrophs.

Cyanobacteria (also known as the Cyanophyceae, Cyanophyta, cyanoprokaryota, blue-green algae or blue-green bacteria) share similar metabolic features with eukaryotic algae and have been named according to the Botanical Code (Kauff and Büdel, 2010). The inclusion of Cyanobacteria in taxonomic schemes of Bacteria was only proposed in 1978 by Stanier et al. (1978), and through time the bacterial taxonomic names have come into conflict with the botanical nomenclature (Oren, 2004; Oren and Garrity, 2014). More than two decades passed before a Note to General Consideration 5 (1999) was published for Cyanobacteria to be included under the rules of the International Committee on Systematic Bacteriology (ICSB)/International Committee on Systematic of Prokaryotes (ICSP) (Tindall, 1999; De Vos and Trüper, 2000; Labeda, 2000). Taxa nomenclature within this group has long been a topic of discussion, but currently there is no consensus (Hoffmann et al., 2005; Oren and Tindall, 2005; Oren et al., 2009; Oren and Ventura, 2017). As a result, more than 50 genera of Cyanobacteria have been described since 2000, and many of them remain unrecognized in the List of Prokaryotic Names with Standing in Nomenclature, LPSN, http://www.bacterio.net (Parte, 2014) or in databases (e.g., NCBI).

The Cyanobacteria form a challenging group for the microbiologists. Their traditional taxonomy based on morphologic traits does not reflect the results of phylogenetic analyses (Rippka et al., 1979; Boone and Castenholz, 2001; Gugger and Hoffmann, 2004; Schirrmeister et al., 2011b; Hugenholtz et al., 2016). The predominance of morphology assembled unrelated Cyanobacteria into polyphyletic species and genera and higher taxonomic categories which require revisions in the future (Komárek et al., 2014). The polyphyly is an indicative of the taxonomic mislabeling of many taxa. The 16S rRNA gene sequences were useful in charting and characterizing microbial communities (Kozlov et al., 2016) but this molecule lack sensitivity for evolutionary changes that occur in ecological dynamics, where microbial diversity is organized by physicochemical parameters (Choudoir et al., 2012; Becraft et al., 2015). Hence, the processes that shape cyanobacterial communities over space and time are less known. A recent study proposed that there should be 170 genera of Cyanobacteria based on 16S rRNA sequences only (Kozlov et al., 2016). Farrant et al. (2016) delineated 121 *Prochlorococcus* and 15 *Synechococcus* ecologically significant taxonomic units (ESTUs) in the global ocean using single-copy *pet*B sequences (encoding cytochrome b6) and environmental cues.

High Throughput Sequencing (HTS) have revolutionized the practice of microbial systematics, providing an informative, reproducible, and portable tool to delineate species, reconstruct their evolutionary history, and infer ecogenomic features (Gevers et al., 2005; Konstantinidis and Tiedje, 2005a,b; Garrity and Oren, 2012; Gribaldo and Brochier-Armanet, 2012; Shih et al., 2013; Sutcliffe et al., 2013; Hugenholtz et al., 2016). This approach allows both cultured (Al-saari et al., 2015; Appolinario et al., 2016) and uncultured microorganisms (Iverson et al., 2012; Brown et al., 2015; Hugerth et al., 2015) to be studied. The latter is especially important because the cyanobacterial cultivation in laboratory is another hurdle in the study of this group of bacteria.

Recommendations that nomenclature should agree with and reflect genomic information were stated during the pre-genomic era (Wayne et al., 1987), due nothing describes an organism better than its genome. Sequence-based methods to delimit prokaryotic species have emerged to define and to improve cut-offs criteria during the genomic era (Gevers et al., 2005; Konstantinidis and Tiedje, 2005a,b; Konstantinidis et al., 2006; Goris et al., 2007; Richter and Rossello-Mora, 2009; Auch et al., 2010a; Thompson et al., 2013a,b; Varghese et al., 2015), demonstrating a greater discriminatory power. Inexorable advances in methodologies will incorporate genomics into the taxonomy and systematics of the prokaryotes, boosting the credibility of taxonomy in the current post-genomic era (Coenye et al., 2005; Chun and Rainey, 2014). Up-to-date, while several groups have been analyzed through a genomic-wide view (Gupta et al., 2015; Adeolu et al., 2016; Hahnke et al., 2016; Ahn et al., 2017; Amin et al., 2017; Waite et al., 2017), many others have faced hurdles, such as Cyanobacteria. However, a genomic taxonomy approach has successfully been applied to elucidate the taxonomic structure of the two cyanobacterial genera, *Prochlorococcus* and *Synechococcus* (Thompson et al., 2013a; Coutinho et al., 2016a,b). As genomic taxonomy postulates numeric, non-subjective, cut-offs for taxa delimitation, strains were considered to belong to the same species when share at least 98.8% 16S rRNA gene sequence similarity, 95% of AAI, and 70% GGD (Konstantinidis and Tiedje, 2005a; Thompson et al., 2013a,b), while species from the same genus form monophyletic branches (Yarza et al., 2008; Qin et al., 2014). It is in agreement with the concept of species as a discrete, monophyletic and genomically homogeneous population of organisms that can be discriminated from other related populations by means of diagnostic properties (Rossello-Mora and Amann, 2001; Stackenbrandt et al., 2002). The availability of whole-genomes opened the doors for an in-depth knowledge in microbial diversity and ecology, where the entire genomic pool may be applied to understanding the forces that govern community structure. The use of ecogenomic analysis postulates a reliable and scalable approach to delineate species and genera in order to reconstruct their evolution and to draw a global picture of possible ecological determinants (Di Rienzi et al., 2013; Soo et al., 2014; Spang et al., 2015; Thompson et al., 2015; Anantharaman et al., 2016; Garrity, 2016; Hug et al., 2016; Hugenholtz et al., 2016). Our hypothesis is that a phylogenomic framework will reflect ecologic groups found in nature.

To test this hypothesis, we first established a phylogenomic framework, using genomic signatures (i.e., a tree based on conserved gene sequences, average amino acid identity, and genome-to-genome distance), with the circumscription of species and genera. We then classified the genomes in three major groups according to their ecological traits as inferred through metagenomics and environmental metadata. Finally, we correlated the three disclosed ecogenomic groups (i. Low Temperature; ii. Low Temperature Copiotroph; and iii. High Temperature Oligotroph) with the circumscribed species and genera. We observed that the taxonomic delineation of species and genera is coherent with the ecogenomic groups.

## Materials and methods

### Genome election

Cyanobacterial genomes publicly available in January 2016 were retrieved from RefSeq (NCBI Reference Sequence Database), GenBank and GEBA (Genomic Encyclopedia of Bacteria and Archaea) databases. Genome completeness was assessed with CheckM (Parks et al., 2015), and the genomes that were at least 90% complete and assembled in < 500 contigs were used for further analyses. Ninety-nine genomes were selected based on that criterion, and they are listed in Table 1 (additional information on Table S2).

**Table 1:** Details of all cyanobacterial genomes included in this study.

**Bacterial Strain^a^**	**Strain^a^**	**NCBI or JGI Reference Sequence**	**New genus proposal**	**New species proposal**	**Habitat**	**Type source/Place**	**# Contigs^Δ^**	**Lenght (Mbp)^^Δ^^**	**% mol GC^^Δ^^**	**# CDS**	Completeness^*^	**Carboxysome**
*Anabaena cylindrica*	PCC 7122^T^	NC_019771.1			Freshwater	Cambridge, UK	7	7.06	38.79	6,182	99.44	β
*Anabaena* sp.	PCC 7108	NZ_AJWF00000000.1		*A. mossi*	Marine (coastal)	Intertidal zone, Moss Beach, CA, USA	3	5.9	38.78	5,169	99.63	β
*Arthrospira platensis*	C1^b^	NZ_CM001632.1		*A. sesilensis*	Freshwater	Alkaline salt lakes	63	6.09	44.69	4,852	99.71	β
*Arthrospira platensis*	NIES-39	NC_016640.1			Freshwater	Alkaline salt lakes	1	6.78	44.27	6,676	99.13	β
*Arthrospira platensis*	Paraca	NZ_ACSK00000000.3			Freshwater	Alkaline salt lakes	239	6.49	44.31	5,436	99.34	β
*Arthrospira* sp.	PCC 8005	NZ_FO818640.1		*A. nitrilium*	Unknown	Unknown	119	6.27	44.7	5,171	99.93	β
*Calotrix* sp.	PCC 7103	NZ_ALVJ00000000.1		*C. wisconsii*	Freshwater	Crawford Co., Wisconsin, USA	12	11.58	38.55	9,371	99.39	β
*Chamaesiphon minutus*	PCC 6605	NC_019697.1			Freshwater	Berkeley, CA, USA	1	6.28	45.73	5,956	99.48	β
*Chroococcidiopsis thermalis*	PCC 7203^T^	NC_019695.1			Soil	Greifswald, Germany	3	6.68	44.47	5,618	99.63	β
*Coleofasciculus chthonoplastes*	PCC 7420^cT^	NZ_ABRS00000000.1			Marine (coastal)	Salt marsh in Woods Hole, Massachusetts, USA	142	8.65	45.43	7,100	99.37	β
*Crinalium epipsammum*	PCC 9333	NC_019753.1			NA	NA	1	5.31	40.16	5,002	99.48	β
*Cyanobacterium*	ESFC-1	NZ_ARCP00000000.1	*Cyclospexia*	*C. valenium*	Marine (coastal)	Extremophylic mat communities, Elkhorn Slough estuary, CA, USA	52	5.62	46.51	4,857	99.59	β
*Cyanobacterium*	JSC-12	NZ_CM001633.1	*Tapinonema*	*T. coecalium*	Freshwater	NA	20	5.52	47.49	5,024	99.29	β
*Cyanobacterium stanieri*	PCC 7202^T^	CP003940.1	*Geminocystis*	*G. stanieri*	Freshwater	Thermal springs, alkaline pod	1	3.16	38.66	2,886	99.52	β
*Cylindrospermum stagnale*	PCC 7417^T^	NC_019757.1			Soil	Stockholm, Sweden	4	7.61	42.2	6,127	99.78	β
*Dactylococcopsis salina*	PCC 8305^T^	NC_019780.1			Freshwater	Solar Lake, Israel	1	3.78	42.44	3,412	99.55	β
*Fischerella* sp.	JSC-11	NZ_AGIZ00000000.1		*F. sesquitii*	NA	NA	34	5.38	41.05	4,627	99.76	β
*Fischerella* sp.	PCC 9339	NZ_ALVS00000000.1		*F. hapalii*	NA	NA	13	8	40.16	6,720	99.76	β
*Fischerella* sp.	PCC 9431	ALVX00000000.1		*F. welwii*	NA	NA	8	7.16	40.19	6,104	99.76	β
*Fischerella* sp.	PCC 9605	NZ_ALVT00000000.1		*F. peptidasii*	Soil	Limestone, Jerucham, Har Rahama, Israel	12	8.08	42.61	7,060	100	β
*Geitlerinema* sp.	PCC 7105	NZ_ANFQ00000000.1		*G. catellasis*	NA	USA	8	6.15	51.59	4,735	93.75	β
*Geitlerinema* sp.	PCC 7407	NC_019703.1	*Pseudogeitlerinema*	*P. shalloid*	Unknown	Unknown	1	4.68	58.46	3,727	99.87	β
*Geminocystis herdmanii*	PCC 6308^T^	NZ_ALVO00000000.1			Freshwater	Lake near Madison, Wisconsin, USA	1	4.26	34.28	3,887	99.78	β
*Gloeocapsa* sp.	PCC 7428	NC_019745.1	*Rotundosa*	*R. thermolimnetic*	Thermal - Freshwater	Moderate hot spring	1	5.43	43.27	5,254	99.78	β
*Gloeocapsa* sp.	PCC 73106	NZ_ALVY00000000.1		*G. sphagnus*	Freshwater	Sphagnum bog, Switzerland	228	4.025	41.11	3,704	98.84	β
*Halothece* sp.	PCC 7418	NC_019779.1	*Dactylococcopsis*	*D. halotolerans*	Freshwater	Solar Lake, Israel	1	4.18	42.92	3,663	99.48	β
*Leptolyngbya boryana*	PCC 6306^T^	NZ_ALVM00000000.1			Freshwater	Lake near Madison, Wisconsin, USA	5	7.26	47.02	6,827	99.41	β
*Leptolyngbya* sp.	PCC 7104^d^	NZ_ALVP00000000.1	*Allonema*	*A. longislandicus*	Marine (coastal)	Rock at shoreline, Montauk Point, Long Island, NY, USA	2	6.89	57.69	6,414	99.18	β
*Leptolyngbya* sp.	PCC 7375	NZ_ALVN00000000.1	*Adonisia*	*A. splendidus*	Marine (coastal)	Plankton, Woods Hole, Massachusetts, USA	5	9.42	47.62	8,366	99.73	β
*Leptolyngbya* sp.	PCC 7376	NC_019683.1	*Enugrolinea*	*E. bermudensis*	Marine (coastal)	Limestone, Crystal Cave, Bermuda	1	5.12	43.87	4,601	99.42	β
*Leptolyngbya* sp.	PCC 6406	NZ_ALVV00000000.2	*Euryforis*	*E. eilemai*	Freshwater	California, USA	3	5.77	55.18	5,156	98.64	β
*Lyngbya aestuarii*	BL-J	NZ_AUZM00000000.1			NA	NA	432	6.87	41.16	5,597	99.74	β
*Lyngbya confervoides*	BDU	NZ_JTHE00000000.1	*Rheamaris*	*R. confervoides*	Marine	India	298	8.79	55.63	8,370	99.34	β
*Lyngbya* sp.	PCC 8106^e^	NZ_AAVU00000000.1		*L. limosa*	Marine (coastal)	NA	110	7.03	41.11	5,854	99.3	β
*Microcoleus* sp.	PCC 7113	NC_019738.1	*Allocoleopsis*	*A. franciscanus*	Soil	San Francisco, California, USA	1	7.47	46.21	6,734	99.56	β
*Microcoleus vaginatus*	FGP-2	NZ_AFJC00000000.1	*Microcoleus*	*M. vaginatus*	Soil	Canyonlands National Park, UT, USA	40	6.69	46.04	5,519	99.67	β
*Moorea producens*	3L^fT^	NZ_AEPQ00000000.1			NA	NA	287	8.38	43.68	6,979	98.56	β
*Nostoc* sp.	PCC 7107	NC_019676.1	*Nostoc*	*N. reyesii*	Freshwater	Point Reyes Peninsula, California, USA	1	6.32	40.36	5,200	99.26	β
*Nostoc* sp.	PCC 7524	NC_019684.1	*Nostoc*	*N. amparaii*	Freshwater	Hot spring, Amparai District, Maha Oya, Sri Lanka	3	6.71	41.53	5,326	99.33	β
*Oscillatoria acuminata*	PCC 6304^T^	NC_019693.1			Soil	NA	1	7.68	47.6	6,004	99.71	β
*Oscillatoria nigroviridis*	PCC 7112	NC_019729.1	*Microcoleus*	*M. nigroviridis*	Soil	USA	1	7.47	45.87	6,925	99.78	β
*Oscillatoria* sp.	PCC 10802	NZ_ANKO00000000.1	*Somacatellium*	*S. hydroxylic*	NA	NA	9	8.59	54.1	7,012	100	β
*Oscillatoria* sp.	PCC 6506	NZ_CACA00000000.1	*Toxinema*	*T. oscillati*	NA	NA	377	6.67	43.4	6,007	99.12	β
*Oscillatoria* sp.	PCC 6407^g^	NZ_ALVI00000000.1	*Toxinema*	*T. oscillati*	Freshwater	NA	12	6.89	43.43	5,693	99.56	β
*Parasynechococcus africanus*	CC9605^T^^•^	NC_007516			Marine	California current, Pacific, oligotrophic, 51 m	1	2.51	59.2	2,583	99.73	α
*Parasynechococcus chillensis*	CC9902^T^^•^	NC_007513			Marine	California current, Pacific, oligotrophic, 5 m	1	2.23	54.2	2,289	99.46	α
*Parasynechococcus marearabicus*	WH8109^T^^•^	ACNY00000000.1			Marine	Sargasso Sea	1	2.12	60.1	2,661	99.32	α
*Parasynechococcus marenigrum*	WH8102^T^^•^	NC_005070.1			Marine	Sargasso Sea	1	2.43	59.4	2,461	99.46	α
*Parasynechococcus nordiatlanticus*	BL107^T^^•^	NZ_DS022298.1			Marine	Blanes Bay, Mediterranean Sea, 1,800 m	1	2.29	54.2	2,322	99.46	α
*Parasynechococcus benguelii*	CC9311^T^^•^	NC_008319.1	*Pseudosynechococcus*	*P. benguelii*	Marine	California current, Pacific, coastal, 95 m	1	2.61	52.4	2,627	99.73	α
*Parasynechococcus equatorialis*	RS9917^T^^•^	NZ_CH724158.1	*Pseudosynechococcus*	*P. equatorialis*	Marine	Gulf of Aqaba, Red Sea, 10 m	1	2.58	64.4	2,575	99.46	α
*Parasynechococcus gyrus*	RS9916^T^^•^	NZ_DS022299.1	*Pseudosynechococcus*	*P. gyrus*	Marine	Gulf of Aqaba, Red Sea, 10 m	1	2.66	59.8	2,603	99.73	α
*Parasynechococcus pacificus*	WH7803^T^^•^	NC_009481	*Pseudosynechococcus*	*P. pacificus*	Marine	Sargasso Sea, 25 m	1	2.37	60.2	2,439	99.18	α
*Parasynechococcus subtropicalis*	WH7805^T^^•^	NZ_CH724168.1	*Pseudosynechococcus*	*P. subtropicalis*	Marine	Sargasso Sea	3	2.63	57.6	2,595	99.73	α
*Parasynechococcus sudipacificus*	WH8016^T^^•^	AGIK00000000.1	*Pseudosynechococcus*	*P. sudipacificus*	Marine	Woods Hole, MA, USA	16	2.69	54.1	2,990	99.18	α
*Parasynechococcus antarcticus*	WH5701^T^^•^	NZ_CH724159–NZ_CH724167	*Regnicoccus*	*R. antarcticus*	Marine	Long Island Sound, Connecticut, USA	116	3.28	65.4	2,917	99.46	α
*Parasynechococcus indicus*	CB0205^•^	NZ_ADXM00000000.1	*Magnicoccus*	*M. indicus*	Marine	Chesapeake Bay, Baltimore, Maryland, USA	78	2.43	63	2,473	99.18	α
*Parasynechococcus sudiatlanticus*	CB0101^T^^•^	NZ_ADXL00000000.1	*Magnicoccus*	*M. sudiatlanticus*	Marine	Chesapeake Bay, Baltimore, Maryland, USA	94	2.69	64.2	2,757	99.73	α
*Parasynechococcus mediterranei*	RCC307^T^^•^	NC_009482.1	*Inmanicoccus*	*I. mediterranei*	Marine	Mediterranean Sea, 15 m	1	2.22	60.8	2,348	99.64	α
*Planktothrix agardhii*	NIVA-CYA 126/8	NZ_CM002803.1		*P. stereotis*	Freshwater	NA	13	5.04	39.57	4,188	100	β
*Planktothrix agardhii*	NIVA-CYA 15	NZ_AVFS00000000.1			Freshwater	NA	238	5.38	39.48	4,606	100	β
*Planktothrix agardhii*	NIVA-CYA 56/3	NZ_AVFY00000000.1			Freshwater	NA	185	5.48	39.48	4,674	99.78	β
*Planktothrix mougeotii*	NIVA-CYA 405	NZ_AVFU00000000.1		*P. agardhii*	Freshwater	NA	240	5.46	39.47	4,697	99.56	β
*Planktothrix prolifica*	NIVA-CYA 406	NZ_AVFV00000000.1		*P. agardhii*	Freshwater	NA	375	5.62	39.51	4,873	100	β
*Planktothrix prolifica*	NIVA-CYA 540	NZ_AVFX00000000.1		*P. agardhii*	Freshwater	NA	157	5.5	39.48	4,710	99.78	β
*Planktothrix prolifica*	NIVA-CYA 98	NZ_AVFZ00000000.1		*P. agardhii*	Freshwater	NA	346	5.61	39.52	4,862	99.78	β
*Planktothrix rubescens*	NIVA-CYA 407	NZ_AVFW00000000.1		*P. agardhii*	Freshwater	NA	219	5.39	39.46	4,658	100	β
*Pleurocapsa* sp.	PCC 7319	NC_019689.1		*P. penascus*	Marine (coastal)	Arizona Station, Gulf of California, Puerto Penasco, Mexico	10	7.38	38.74	4,516	99.56	β
*Pleurocapsa* sp.	PCC 7319	NC_019689.1		*P. penascus*	Marine (coastal)	Arizona Station, Gulf of California, Puerto Penasco, Mexico	10	7.38	38.74	4,516	99.56	β
*Prochlorococcus chisholmii*	AS9601^T^^○^	NC_008816.1	*Eurycolium*	*E. chisholmii*	Marine	Arabian Sea, 50 m	1	1.66	31.32	1,769	99.64	α
*Prochlorococcus marinus*	CCMP1986	NC_005072.1	*Eurycolium*	E. marinus	Marine	Mediterranean Sea, 5 m	1	1.65	30.8	1,777	99.46	α
*Prochlorococcus neptunius*	MIT9312^T^^○^	NC_007577.1	*Eurycolium*	*E. neptunius*	Marine	Gulf Stream, 135 m	1	1.7	31.21	1,815	99.73	α
*Prochlorococcus nereus*	MIT9202^T^^○^	NZ_ACDW00000000.1	*Eurycolium*	*E. nereus*	Marine	South Pacific, 79 m	1	1.69	31.1	1,795	98.78	α
*Prochlorococcus nereus*	MIT9215^○^	NC_009840.1	*Eurycolium*	*E. nereus*	Marine	Equatorial Pacific, surface	1	1.73	31.15	1,840	99.73	α
*Prochlorococcus ponticus*	MIT9301^T^^○^	NC_009091.1	*Eurycolium*	*E. ponticus*	Marine	Sargasso Sea, 90 m	1	1.64	31.34	1,774	99.46	α
*Prochlorococcus tetisii*	MIT9515^T^^○^	NC_008817.1	*Eurycolium*	*E. tetisii*	Marine	Equatorial Pacific, 15 m	1	1.7	30.79	1,784	100	α
*Prochlorococcus proteus*	NATL1A^○^	NC_008819.1	*Prolificoccus*	*P. proteus*	Marine	Northern Atlantic, 30 m	1	1.86	34.98	2,204	99.73	α
*Prochlorococcus proteus*	NATL2A^T^^○^	NC_007335.2	*Prolificoccus*	*P. proteus*	Marine	Northern Atlantic, 10 m	1	1.84	35.12	1,930	99.45	α
*Prochlorococcus marinus*	CCMP1375^T^	NC_005042.1			Marine	Sargasso Sea, 120 m	1	1.75	36.44	1,883	100	α
*Prochlorococcus ceticus*	MIT9211^T^	NC_009976.1		*P. ceticus*	Marine	Equatorial Pacific, 83 m	1	1.68	38.01	1,748	99.73	α
*Prochlorococcus swingsii*	MIT9303^○^	NC_008820.1	*Thaumococcus*	*T. swingsii*	Marine	Sargasso Sea, 100 m	1	2.68	50.01	2,504	100	α
*Prochlorococcus swingsii*	MIT9313^T^^○^	NC_005071.1	*Thaumococcus*	*T. swingsii*	Marine	Gulf Stream, 135 m	1	2.41	50.74	2,339	99.46	α
*Pseudanabaena biceps*	PCC 7429	NZ_ALWB00000000.1			Freshwater	NA	464	5.47	43.18	4,774	99.29	β
*Pseudanabaena* sp.	PCC 7367	NC_019701.1	*Leptolatis*	*L. gracile*	Marine (coastal)	Intertidal zone, Mexico	1	4.55	46.31	3,960	98.23	β
*Pseudoanabaena* sp.	PCC 6802	ALVK00000000.1	*Paraleptovivax*	*P. allomegium*	Freshwater	California, USA	6	5.62	47.83	5,363	99.76	β
*Rivularia* sp.	PCC 7116	NC_019678.1		*R. bajacalifornii*	Marine (coastal)	La Paz, Baja California Sur, Mexico	3	8.72	37.53	6,612	99.78	β
*Spirulina subsalsa*	PCC 9445	NZ_ALVR00000000.1	*Paraspirulina*	*P. subsalsa*	NA	NA	10	5.32	47.39	4,580	99.56	β
*Stanieria cyanosphaera*	PCC 7437^T^	NC_019748.1			Freshwater	Havana, Cuba	6	5.54	36.22	4,895	99.56	β
*Synechococcus elongatus*	PCC 6301^T^	NC_006576.1			Freshwater	NA	1	2.7	55.5	2,576	99.73	β
*Synechococcus elongatus*	PCC 7942	NC_007604.1			Freshwater	NA	2	2.74	55.46	2,655	100	β
*Synechococcus springii*	JA23Ba213^T^^•^	NC_007776	*Leptococcus*	*L. springii*	Thermal-Freshwater	Octopus Spring, Yellowstone Park, USA	1	3.05	58.5	3,064	100	β
*Synechococcus yellowstonii*	JA33Ab^T^^•^	NC_007775.1	*Leptococcus*	*L. yellowstonii*	Thermal-Freshwater	Octopus Spring, Yellowstone Park, USA	1	2.93	60.2	3,036	100	β
*Synechococcus californii*	PCC 6312^T^^•^	NC_019680.1	*Stenotopis*	*S. californii*	Freshwater	California, USA	2	3.72	48.49	3,795	99.29	β
*Synechococcus euryhalinus*	PCC 7002^T^^•^	NC_010475.1	*Enugrolinea*	*E. euryhalinus*	Unknown	Unknown	7	3.41	49.16	3,121	100	β
*Synechococcus mexicanus*	PCC 7335^T^^•^	ABRV00000000.1	*Coccusdissimilis*	*C. mexicanus*	Marine (coastal)	Snail shell, intertidal zone, Puerto Penasco, Mexico	11	5.97	48.2	5,702	98.91	β
*Synechococcus berkleyi*	PCC 7336^T^^•^	ALWC00000000.1	*Brevicoccus*	*B. berkleyi*	Marine (coastal)	Sea Water Tank, Berkeley University, CA, USA	1	5.07	53.7	5,093	100	β
*Synechococcus bogii*	PCC 7502^T^^•^	CP003594.1	*Leptovivax*	*L. bogii*	Sphagnum bog (peat bog)	NA	3	3.58	40.6	3,703	99.76	β
*Synechocystis* sp.	PCC 7509	ALVU00000000.2	*Doliumcoccus*	*D. switzii*	Soil	Rock scraping, Switzerland	4	4.9	41.67	4,859	99.67	β
*Trichodesmium erythraeum*	IMS101^T^	NC_008312.1			Marine (coastal)	NA	1	7.75	34.14	4,358	99.71	β
*Xenococcus* sp.	PCC 7305	NZ_ALVZ00000000.1		*X. lajollai*	Marine (coastal)	Aquarium, La Jolla, CA, USA	234	5.92	39.68	4,992	99.78	β
*Gloeobacter violaceum*	PCC 7421^h^	NC_005125.1			Soil	Calcareous (chalky) rock, Switzerland	1	4.66	62	4,511	99.15	β

*Ecological and molecular features were indicated, such as environment sampling, as well as number of contigs, genome size, GC % content, completeness score, and carboxysome type. The following classifications for detailed for comparison: NCBI (order and family), numeral identification and genera according to Kozlov et al. (2016), and identification based in the database CyanoType v.1 (Ramos et al., 2017). Type strains or type species are indicated with overwritten T at the end of the name*.

a *Cyanobacterial genomes used in Komárek et al. (2014) paper and available at public database in January 2016 were retrieved for this study*.

b *Arthrospira platensis is also called Spirulina platensis*.

c *Coleofasciculus chthonoplastes PCC 7420 is also called Microcoleus chthonoplastes PCC 7420*.

d *Leptolyngbya sp. PCC 7104 is also called Nodosilinea nodulosa PCC 7104*.

e *Lyngbya aestuarii PCC8106 is also called L. aestuarii CCY9616, and even the former name Oscillatoria limosa PCC8106*.

f *Moorea producens 3L is also called Moorea producta 3L*.

g *Oscillatoria sp. PCC 6407 is also called Kamptonema formosum PCC 6407, and even O. formosa PCC 6407*.

h *Outgroup used in the phylogenetic analysis*.

• *New taxonomic identification proposed by Coutinho et al. (2016a,b)*.

○ New taxonomic classification proposed by Thompson et al. (2013a)

Δ *Number of contigs, total length and GC content values were obtained using QUAST tool*.

* *Values using CheckM tool*.

### Annotation and genomic taxonomy

All genomes were annotated using Prokka version 1.11 (Seemann, 2014), with default settings, in order to avoid any possible bias. Genomic taxonomy of the ninety-nine cyanobacterial genomes was performed according to Thompson et al. (2013a) and Coutinho et al. (2016a) and are briefly described here. Average Amino acid Identity (AAI) and Genome-to-Genome Distance (GGD) were calculated as described previously (Konstantinidis and Tiedje, 2005a; Auch et al., 2010a,b; Meier-Kolthoff et al., 2013). GGD were calculated using the Genome-to-Genome Distance Calculator tool, version 2.1 under recommended settings (Meier-Kolthoff et al., 2013; http://ggdc.dsmz.de/), whereas AAI values were carried out through GenTaxo as previously described (Coutinho et al., 2016a). The species cut-offs delimitation were ≥95% AAI and ≥70% GGD, and ≥70% AAI for genus delimitation.

The Manhattan distances were calculated based on the percentage AAI values of every genome (genome-genome matrix) and was used as the input for making the hierarchical clustering using the hclust() function in R (R Development Core Team, 2011). This distance is able to indicate how far/close the genomes are located from each other. The heatmap was produced by heatmap.2 {gplots} package in R, with background color of each panel mapping to percentage AAI values.

### Phylogenetic analysis

To establish the phylogenetic structure of the phylum Cyanobacteria, phylogenetic trees were constructed using the 16S rRNA gene sequences and the concatenated alignments of a set of conserved genes, most of which encode ribosomal proteins.

#### Ribosomal RNA sequences

The small subunit ribosomal RNA (16S rRNA) sequences from all cyanobacterial strains for which whole genome sequence data are publicly available (exception see below, thus *N* = 97), as well as 16S rRNA gene sequences from additional type-strains available (*N* = 14) were all analyzed. The sequences were retrieved from the ARB SILVA database (Pruesse et al., 2007; Quast et al., 2013). Whenever sequences were not available, they were retrieved directly from the genomes using RNammer 1.2 Server (Lagesen et al., 2007). Sequences were aligned through MUSCLE v. 3.8 (Edgar, 2004), with default settings, and Gblocks 0.91b (Castresana, 2000; Talavera and Castresana, 2007) was used for alignment curation. Using MEGA 6 (Tamura et al., 2013), best-fitting nucleic acid substitution models were calculated through the MLModelTest feature. Models were ranked based on their Bayesian Information Criterion (BIC) scores as described by Tamura et al. (2013). The model with the lowest BIC score was selected and used for further phylogenetic analysis. The phylogenetic inference was obtained using the Maximum Likelihood method based on the Kimura 2 parameter method with the Gamma distributed rate variation (K2+G) as the nucleotide substitution model, which was estimated from the data. The support branches of tree topology were checked by 1,000 bootstrap replicates. The 16S rRNA gene alignments were used to estimate the degree of genetic distance between strains through the Tajima-Nei method (Tajima and Nei, 1984).

*Gloeobacter violaceus* PCC 7421 was set as the outgroup in both trees. Trees were visualized with FigTree, version 1.4.2 (Rambaut, 2015). Due to incomplete or partial sequences, *Synechococcus* sp. CB0101 was omitted from these analyses. *Planktothrix mougeotii* NIVA-CYA 405 as well as *Planktothrix prolifica* NIVA-CYA 540 were not included in the phylogenetic analyses because 16S rRNA sequences are not currently available for these strains (and not retrievable from their genomes).

The type-strains or the type-species of each taxa were included in the 16S phylogenetic tree to confirm the phylogenetic relatedness of the cyanobacterial genomes. Designations of type strain or type species were not available for *Chaemaesiphon minutus* PCC6605, *Pleurocapsa* sp. PCC7319, *Rivularia* sp. PCC7116, *Synechocystis* sp. PCC7509, *Trichodesmium erythraeum* IMS01, *Xenococcus* sp. PCC7305, cyanobacterium ESFC-1, and cyanobacterium JSC-12. *Geitlerinema* sp. PCC7105 is the reference strain for marine species of *Geitlerinema*, and PCC73106 is the reference strain for *Gloeocapsa* (Sarma, 2012).

#### Conserved marker genes

A tree was generated using 31 conserved gene sequences previously validated as phylogenetic markers for (cyano) bacteria (Wu and Eisen, 2008, and recently used by Shih et al., 2013 and Komárek et al., 2014). The sequences of these proteins were mined using the AutoMated Phylogenomic infeRence Application—AMPHORA2 tool (Wu and Scott, 2012), through default settings for the Bacteria option, and with a cut-off value of 1.e−10. Individual alignments were performed for each of the 31 gene sets through MUSCLE v. 3.8 with default settings (Edgar, 2004). All alignments were then concatenated. Only genomes which present all the set of conserved genes were used in the phylogenetic analysis. A Maximum Likelihood tree was constructed using RaxML v. 7 (Stamatakis, 2006) and the Dayhoff+G likelihood model. One thousand bootstrap replications were calculated to evaluate the relative support of the branches. Trees were visualized with FigTree, version 1.4.2 (Rambaut, 2015).

### Abundance of cyanobacterial genomes across aquatic environments and ecological correlations

Marine and freshwater metagenomes were retrieved to determine the abundance of ninety-nine cyanobacterial genomes across the Earth. A set of 191 marine metagenomes from the Tara Ocean project were retrieved for analysis along with their associated metadata (Sunagawa et al., 2015). Sample-associated environmental data were inferred across multiple depths at global scale of Tara's metagenomics sampling: (i) surface water layer (5 m, s.d. = 0); and (ii) subsurface layer, including deep chlorophyll maximum zone (71 m, s.d. = 41 m) and mesopelagic zone (600 m, s.d. = 220 m) (Sunagawa et al., 2015). Eight freshwater metagenomes were retrieved for analysis from the Caatinga biome microbial community project along with their associated metadata (Lopes et al., 2016).

Metagenome reads were mapped to a database containing the ninety-nine analyzed cyanobacterial genomes through Bowtie2 (Langmead and Salzberg, 2012) using -*very*-*sensitive*-*local* and -*a* options. Abundance of genomes across samples was calculated based on the number of mapped reads as described by Iverson et al. (2012). Metagenomes were compared based on the relative abundances of the ninety-nine analyzed genomes within them using non-metric multidimensional scaling (NMDS).

Spearman correlation coefficients (R, or Spearman's rho) were calculated for the abundance of each genome and the levels of measured environmental parameters across samples. Next, a dissimilarity matrix of Manhattan distances was calculated based on the Spearman correlation values of every genome. All correlations were used by this analysis regardless of the corrected *p*-value, as non-significant correlations are still ecologically informative as they indicate weak associations between microorganisms and environmental parameters. Finally, this dissimilarity matrix was used as input for hierarchical clustering using the complete linkage method within the hclust() function in R. The resulting dendrogram was visually inspected to define groups (i.e., ecogenomic groups) of organisms with similar correlation patterns which were named based on the main correlated feature.

The classification reassessment was made integrating the results of genomic taxonomy, phylogenomic analysis and ecogenomic signals through an accurately comparison.

## Results

### Phylogenomic framework reconstruction

**Figure 1:**
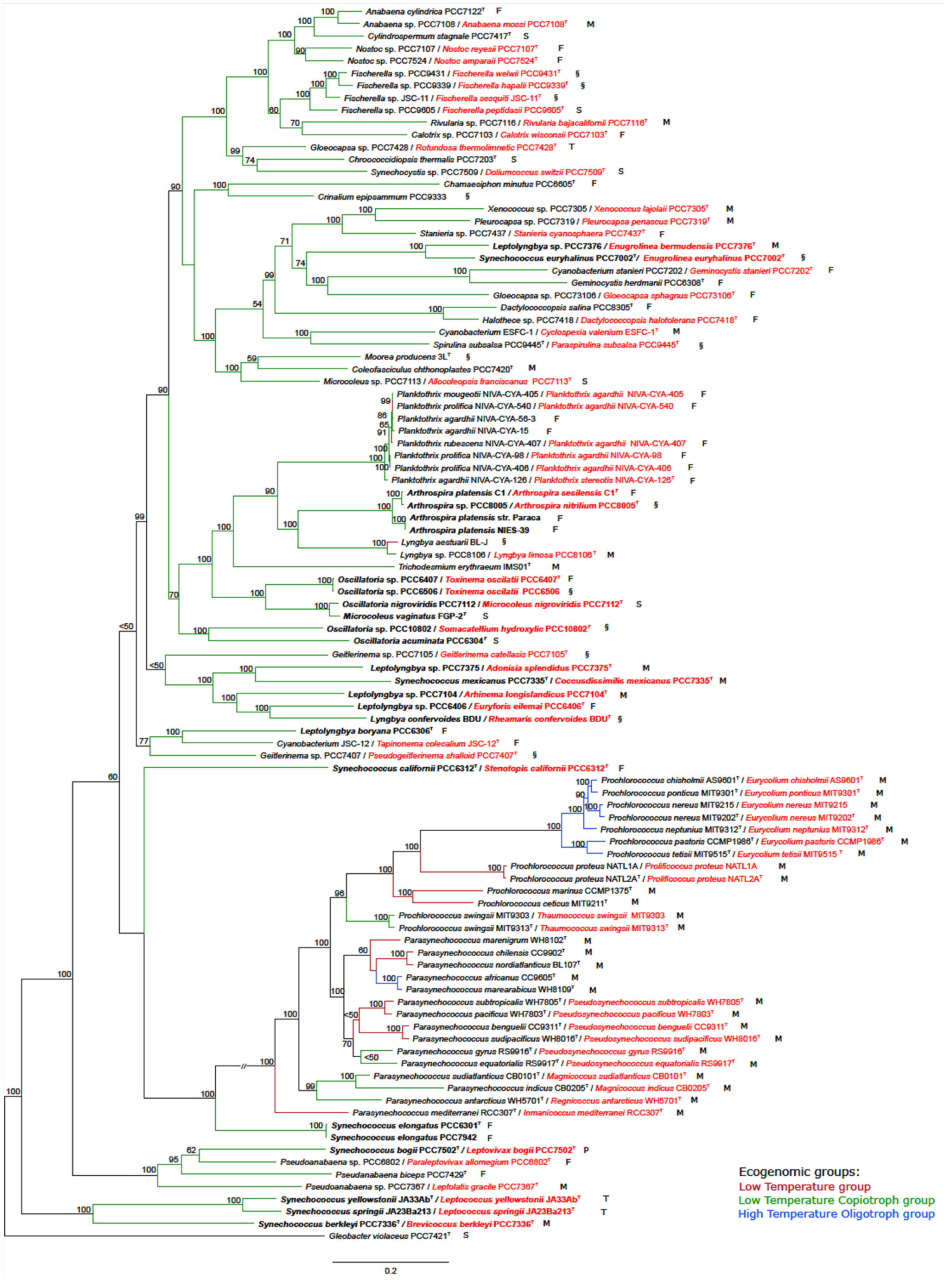
Phylogenomic tree of the Cyanobacteria phylum with the proposed new names. Tree construction was performed using 100 genomes (ninety-nine used in this study plus the outgroup), based on a set of conserved marker genes. The numbers at the nodes indicate bootstrap values as percentages greater than 50%. Bootstrap tests were conducted with 1,000 replicates. The unit of measure for the scale bars is the number of nucleotide substitutions per site. The *Gloeobacter violaceus* PCC 7421 sequence was designated as outgroup. Capital letters indicate environmental source: F, freshwater; M, marine; P, peat bog (sphagnum); S, soil; T, thermal; and §, other habitat. New names are highlighted in red. Overwritten T indicates type strain or type species. Ecogenomic groups are depicted in different colors as indicated in the legend: Low Temperature group; Low Temperature Copiotroph group; and High Temperature Oligotroph group. Cases depicted in the Results section are in bold.

The tree based on conserved marker genes (Figure 1) revealed the topology with the presence of well-defined nodes in general with bootstrap support values greater than 50% over 1,000 replicates. The phylogenomic tree (Figure 1) gave a higher resolution than the 16S rRNA phylogenetic analysis (Figure S1 and Table S1), in the means that strains were better discriminated in the conserved marker genes tree (e.g., *Parasynechococcus* group, Figure 1 and Figure S1). The species assignations were considered correct when organisms located on the same phylogenetic branch as the corresponding type strains or type species presented the 16S rRNA sequence similarity higher than 98.8%, such as *Crinalium epipsammum* SAG22.89^T^ (Figure S1) and *Crinalium epipsammum* PCC9333 (Figure S1 and Figure 1).

### Genomic diversity of cyanobacteria

**Figure 2:**
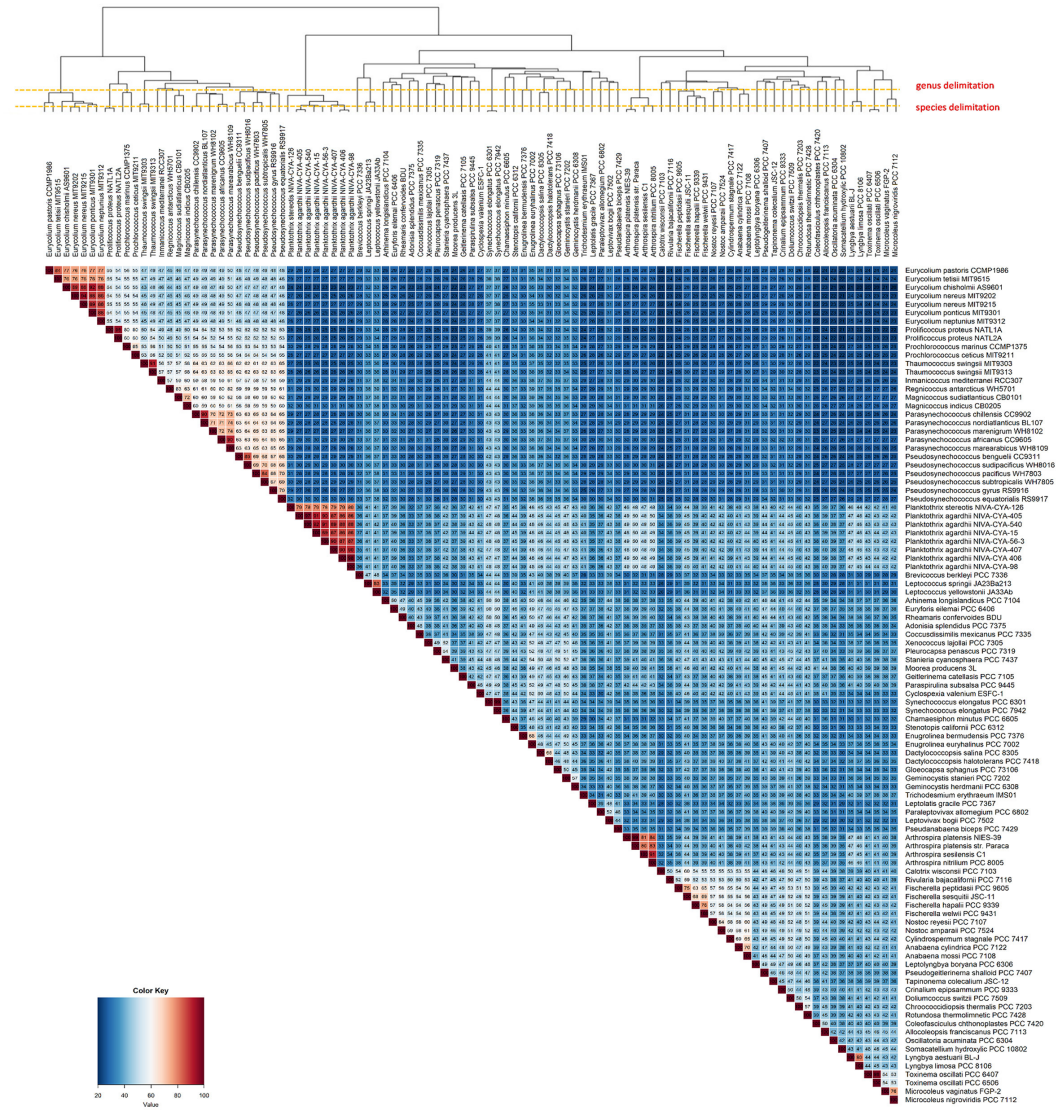
Heatmap displaying the AAI levels between cyanobacterial genomes. The intraspecies limit is assumed as ≥95%, whereas genera delimitation is assumed as ≥70% (dashed lines) AAI. Clustering the genomes by AAI similarity was done using a hierarchical clustering method in R (hclust), based on Manhattan distances. The AAI values are associated with the respective thermal color scale located at the bottom left corner of the figure. The proposed new genera and species names were adopted in this figure.

In total, we found 57 branches corresponding to genera based on the AAI and GGD analyses (Figure 2). The genus and species cut-off delimitation were ≥70% and ≥95% AAI similarity respectively. Thirty-three new genera and 87 species (of which 28 are new species) were circumscribed. From a total of ninety-nine genomes used in this study, 69 were previously classified to the species level, whereas the remaining 30 had incomplete taxonomic classification (i.e., only sp. or unclassified). In total, 13 genera (from a total of 33) and 38 species (from a total of 69) were taxonomically reclassified and/or re-named. Thus, we found that 71 of all analyzed genomes required reassignment at one or more ranks to reconcile existing taxonomic classifications with our new genomic taxonomy (Figure 2 and Figure S1).

Over the next section, we highlight four specific cases to exemplify cyanobacterial taxonomic issues that were resolved through our genome-driven approach (see Figure S2). These cases illustrate how the use of genomic taxonomy in Cyanobacteria provides relevant information (Data Sheet 1, Formal description of new genera and species).

Case I. *Oscillatoria* group. Analysis of the five genomes of *Oscillatoria* distinguished four genera, based on the genomic signatures (i.e., GGD, AAI, 16S, and conserved marker genes tree): (i) *Oscillatoria acuminata* PCC 6304 type strain formed a separate group; (ii) *Oscillatoria* sp. PCC 10802 formed a separate divergent group, corresponding to a new genus named *Somacatellium* (*S. hydroxylic* PCC 10802^T^); (iii) *Oscillatoria nigroviridis* strain PCC 7112 (closest related with *Microcoleus vaginatus* FGP-2 type strain) belongs to the genus *Microcoleus* (*M. nigroviridis* PCC 7112^T^); and (iv) *Oscillatoria* strains PCC 6407 and PCC 6506 formed a new genus named *Toxinema* (*T. oscillati* PCC 6407^T^ and *T. oscillati* PCC 6506).

Case II. *Leptolyngbya* group. The five *Leptolyngbya* strains were polyphyletic, forming different phylogenetic branches. Thus, (i) *Leptolyngbya boryana* PCC 6306^T^ type strain forms a separate group with cyanobacterium JSC-12, while the rest of the *Leptolyngbya* strains cluster apart; (ii) strain PCC 7376 forms a new genus named *Enugrolinea* (*E. bermudensis* PCC 7376^T^); (iii) strain PCC 7375 forms a new genus named *Adonisia* (*A. splendidus* PCC 7375^T^); (iv) strain PCC 7104 forms a new genus named *Allonema* (*A. longislandicus* PCC 7104^T^); and (v) strain PCC 6406 forms a new genus named *Euryforis* (*E. eilemai* PCC 6406^T^).

Case III. *Arthrospira* group. Examination of the four *Arthrospira* strains indicated that (i) *A. platensis* C1 should be considered a new species, named *A. sesilensis* (*A. sesilensis* C1^T^); (ii) strain PCC 8005 belongs to a new species, named *A. nitrilium* (*A. nitrilium* PCC 8005^T^); and (iii) the type strain of *Arthrospira platensis* (PCC 7345) formed a tight cluster along with NIES-39 and Paraca.

Case IV. *Synechococcus* group. The nine *Synechococcus* strains split in (i) *S. elongatus* PCC 6301^T^ type strain forms a separate group with *S. elongatus* PCC 7942; (ii) strain PCC 6312 forms a new genus named *Stenotopis* (*S. californii* PCC 6312^T^); (iii) strain PCC 7335 belongs to a new genus named *Coccusdissimilis* (*C. mexicanus* PCC 7335^T^); (iv) strains JA23Ba213 and JA33Ab formed a new genus named *Leptococcus* (*L. springii* JA23Ba213^T^ and *L. yellostonii* JA33Ab^T^); (v) strain PCC 7336 formed a new genus named *Eurycoccus* (*E. berkleyi* PCC 7336^T^); (vi) strain PCC 7502 belonged to a new genus named *Leptovivax* (*L. bogii* PCC 7502^T^); and (vii) *Synechococcus euryhalinus* PCC 7002 represents a new genus named *Enugrolinea* (*E. euryhalinus* PCC 7002^T^).

### Charting ecological groups of cyanobacteria

**Figure 3:**
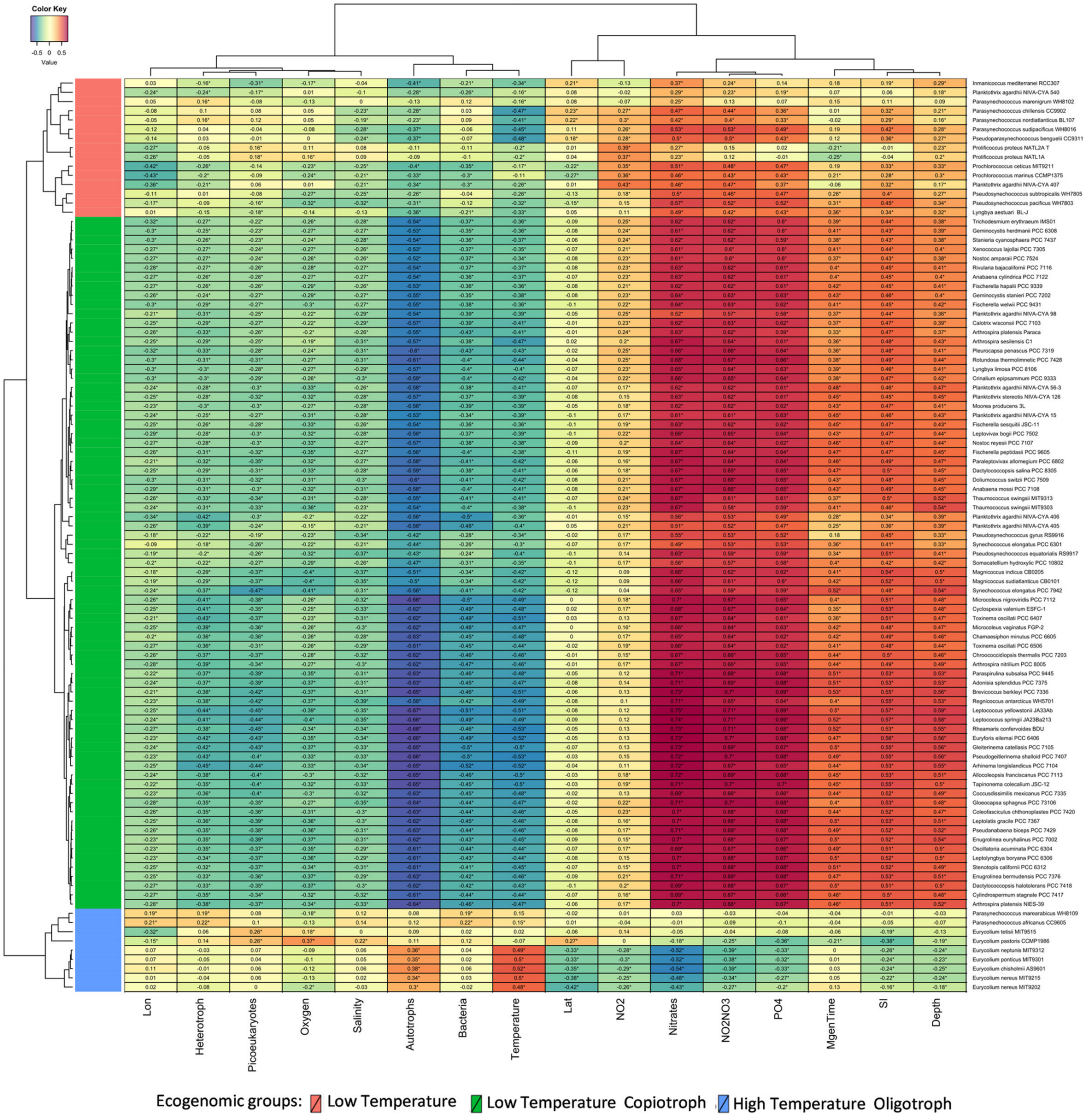
Correlations between Cyanobacteria and environmental variables. Heatmap displays Spearman correlation scores between the abundance of cyanobacterial genomes and measured environmental parameters at *Tara* Ocean sampling sites. Correlations that showed q corrected *p* < 0.05 are marked with stars. Variables were grouped through the complete linkage clustering method using Manhattan distances as input. The proposed new genera and species names were adopted in this figure.

**Figure 4:**
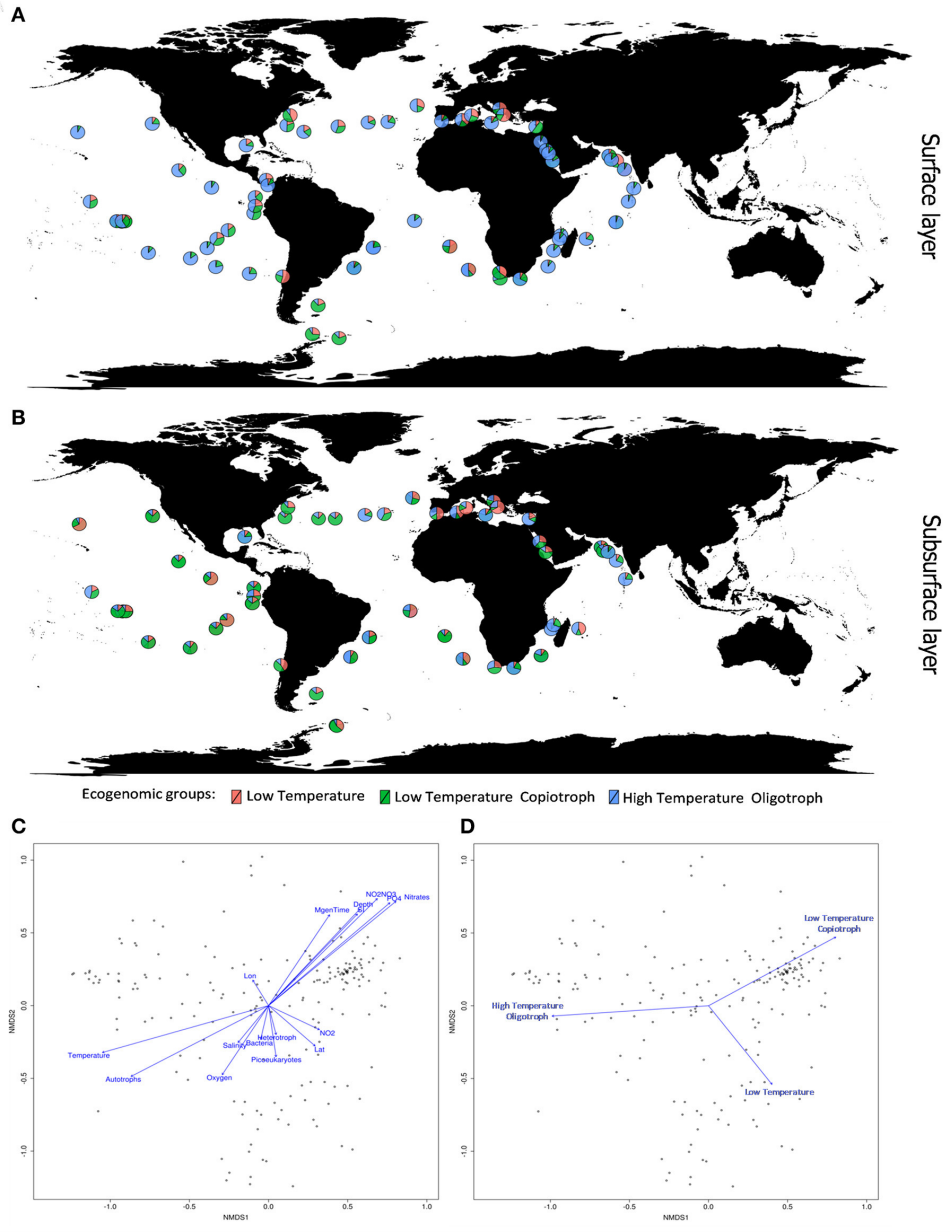
Ecogenomic analysis of Cyanobacteria in global marine environments. **(A)** Distribution of the dominant ecogenomic groups (Low Temperature group; Low Temperature Copiotroph group; and High Temperature Oligotroph) along the *Tara* Ocean transect sampling from surface layer (5 m). **(B)** Distribution of the dominant ecogenomic groups along the *Tara* Ocean transect sampling from subsurface layer (>5 m). **(C)** Non-metric multidimensional scaling (NMDS) analysis of the marine metagenomes and environmental parameters. Ordination plot of physicochemical parameters. Dots indicate the metagenomes samples. Distances were calculated based on the Bray-Curtis Method. NMDS stress value = 0.15. **(D)** Non-metric multidimensional scaling (NMDS) analysis of the marine metagenomes and environmental parameters. Ordination plot of ecogenomic clusters. Dots indicate the metagenomes samples. Distances were calculated based on the Bray-Curtis Method. NMDS stress value = 0.15.

Our phylogenomic analysis was complemented by an ecological characterization of the analyzed strains, providing essential insights into relations between taxonomy, phylogeny, and ecological role (Beiko, 2015). Correlating the relative genome abundances with environmental parameters measured at Tara Oceans samples (Sunagawa et al., 2015) revealed associations between Cyanobacteria and physical, chemical and biological variables of their habitats (Figure 3). The ecogenomic analysis clustered genomes based on their profiles of correlations to environmental parameters. Three major ecogenomic groups were found: (a) Low Temperature; (b) Low Temperature Copiotroph; and (c) High Temperature Oligotroph (Figure 4 and Figure S3). Closely related species of the same genus showed tight associations with environmental parameters, grouped to the same ecogenomic group, such as *Arthrospira sesilensis* C1^T^ and *A. nitrilium* PCC 8005^T^, *Eurycolium pastoris* CCMP1986^T^ and *E. tetisii* MIT9515^T^, and *Pseudosynechococcus subtropicalis* WH7805^T^ and *P. pacificus* WH7803^T^ (Figure 3). In a few cases, closely related species showed different ecogenomic groups (*P. agardhii* NIVA-CYA-407 and *P. agardhii* NIVA-CYA-540 compared to other *Planktothrix* strains, and between *Lyngbya aestuarii* BL-J and *L. limosa* PCC 8106^T^) (Figure 3).

Members of the Low Temperature group were characterized by positive correlations with the concentration of nitrogen and phosphorus sources; weak positive correlations with minimum generation time, silicate and depth; and by negative correlations with temperature, microbial cell abundance, oxygen availability, and salinity (Figures 3, 4). Meanwhile, members of the Low Temperature Copiotroph group were characterized by strong positive correlations with the concentration of nitrogen and phosphorus; positive correlations (stronger than those presented by Low Temperature group) with minimum generation time, silicate and depth; and by negative correlations (also stronger than those presented by Low Temperature group) with temperature, microbial cell abundance (in particular with autotroph cell density), oxygen availability, and salinity (Figures 3, 4). Finally, members of High Temperature Oligotroph group were characterized by negative correlations with the concentration of nitrogen and phosphorus and positive correlations with temperature and autotroph cell abundance (Figures 3, 4).

As suggested by correlation analyses (Figures 4C,D), NMDS revealed the Low Temperature Copiotroph group to be more abundant in cold and eutrophic waters, while the High Temperature Oligotroph group exhibited the opposite pattern and was more abundant in warm and oligotrophic environments (Figures 4A,B). In turn, Low Temperature was more abundant at intermediate conditions between these polar opposites and was shown to be more abundant in samples with higher cell densities and NO_2_ concentrations.

We also investigated the abundance of the ecogenomic groups in freshwater environments. Unfortunately, there is no currently available large-scale dataset of freshwater metagenomes with associated metadata comparable to the Tara Oceans dataset. To define freshwater ecogenomic groups we chose to extrapolate the classification obtained from the analyses of the marine dataset. In freshwater metagenomes, the Low Temperature Copiotroph was the dominant group in all the analyzed samples (Figure S4A). NMDS of freshwater samples suggested that Low Temperature group displayed a preference for higher pH and DOC, nitrite and total nitrogen concentrations whereas the High Temperature Oligotroph group has a preference for habitats with higher concentrations of POC, phosphorus, ammonia and nitrate (Figures S4B,C).

## Discussion

The use of HTS technologies and environmental surveys have allowed studies that link phylogenomics and ecogenomics of Cyanobacteria. High-throughput genome sequence technologies are causing a revolution in microbial diversity studies. Recent studies have obtained dozens of new metagenome-assembled genomes from complex environmental samples (Brown et al., 2015; Hugerth et al., 2015; Almstrand et al., 2016; Haroon et al., 2016; Pinto et al., 2016). The abundance of these genomes across different environments can now be inferred from metagenomics, including their metabolic and ecological potential. It is clear that a new system is required to allow for precise taxonomic identification of these new genomes.

### WGS as the basic unit for cyanobacteria genomic taxonomy (CGT)

Comparative genomic studies allow for identification of sequence groups with high genotypic similarity based on variation in protein coding genes distributed across the genomes. Analyses of environmental metagenomes and microbiomes have shown that microbial communities consist of genotypic clusters of closely related organisms (Farrant et al., 2016). These groups display cohesive environmental associations and dynamics that differentiate them from other groups co-existing in the same environment. In light of new concepts, restlessness is mounting with the inability to define the microbial species itself. Evolution studies on closely related bacteria show rapid and highly variable gene fluxes in evolving microbial genomes, suggesting that extensive gene loss and horizontal gene transfer leading to innovation are the dominant evolutionary processes (Batut et al., 2014; Puigbò et al., 2014). CGT will solve the often-observed issue that even closely related genomes contain high gene content variation, that gives phenotypic variation. CGT is completely adjusting to the genomics era, addressing the needs of its users in microbial ecology and clinical microbiology, in a new paradigm of open access (Beiko, 2015). CGT will provide a predictive operational framework for reliable automated and openly available identification and classification (Thompson et al., 2015).

### Proposals for cyanobacterial taxonomy

A main gap exists and is growing each day between the formal taxonomy of Cyanobacteria and the forest of acronyms and numbers in the different databases. Indeed, the nameless operational taxonomic units (OTUs), strains, isolates and WGS sequences (Beiko, 2015; Kozlov et al., 2016) form the great majority of data in private and public databases. There is a need to re-examine the Cyanobacteria prokaryote species, taking into account all recently developed concepts, e.g., the gene flow unit, OTU, ESTU and Candidate taxonomic unit (CTU) in the context of a pragmatic genome-based taxonomic scheme. The type species or strain can be a culture, DNA or a WGS. The CGT system should maintain all of the existing information, integrating it with new data on DNA, genomes, isolates/strains, cultured and uncultured, “Candidatus” cases and reconstructed genomes from metagenomes (Brown et al., 2015; Hugerth et al., 2015). The international initiatives of GEBA are currently working on determining the WGS of all type strains of known microbial species to shorten this gap (more than eleven thousand genomes).

We strongly recommended that the modern taxonomy should be based on WGS. The enormous amount of unique gene sequences (e.g., 16S rRNA gene) databases should be always compared to the available genome-based phylogeny. Studies focusing on one specific taxa/group cannot be disregarded the phylogenetic analysis for the whole major taxa. It will avoid the inclusion of the previously erroneous taxa on the analysis. Furthermore, the anxiety to give a new name should be reconsidered. Proposes of new taxa where the phylogenetic relationship was not firmly established are frequently found (e.g., Rajaniemi et al., 2005).

### Ecogenomics and the delineation of the ecological niches of cyanobacteria

Correlation analysis allowed us to characterize how the abundance of the analyzed genomes is associated with environmental parameters at both marine and freshwater habitats. These associations shed light on ecological interactions taking place within aquatic habitats that are responsible for delineating the ecological niches of Cyanobacteria. Our results showed that taxonomic affiliation and niche occupancy are coherently linked, i.e., closely related species of the same genus often shared correlation patterns, and consequently were assigned to the same ecogenomic group.

The identification of specific features responsible for defining niche occupancy among these organisms depends on extensive experimental data focusing on both physiological and morphological features, which is outside of our scope. Nevertheless, we speculate that some features are likely playing a role in this process:

1. Transcriptional patterns: The way in which Cyanobacteria regulate gene expression in response to changing environmental conditions is likely to play a role in defining which habitats are better suitable for growth of different species.
2. Nutrient uptake and utilization: Throughout the aquatic environment a myriad of gradients of nutrient abundance are formed (Stocker and Seymour, 2012). The cyanobacterial capacity for uptake and utilization of limiting nutrients (e.g., P, N and Fe) is associated with their ecological niches occupancy (Thompson et al., 2013a; Coutinho et al., 2016b; Farrant et al., 2016). Considering that significant associations were detected between the abundance of the analyzed genomes and the nutrients sources (phosphorus and nitrogen), we assume that the diversity and efficiency of their nutrient transporters plays a major role in defining the cyanobacterial affiliation to the proposed ecogenomic groups.
3. Photosynthetic machinery and efficiency: Cyanobacteria are remarkably diverse when considering their photosynthetic physiology. Species differ with regard their preferred light intensities and wavelengths which affects their photosynthetic efficiency (Moore et al., 1998; Ting et al., 2002). They also can be differentiated regarding their carboxysomes, sub-cellular structures where carbon fixation takes place (Yeates et al., 2008). To our knowledge, no study has consistently compared the photosynthetic yields of all the strains analyzed here, therefore we cannot determine if the proposed ecogenomic groups differ regarding this parameter. Nevertheless, distinctions regarding their requirements for efficient photosynthesis are likely linked to their patterns of niche occupancy.

### Ecogenomics, global changes, and cyanobacterial communities

Over the past two centuries, human development has affected aquatic ecosystems due to nutrient over-enrichment (eutrophication), hydrologic alterations, global warming and ocean acidification. Temperature is one of the most important factors determining the taxonomic composition of marine microbial communities (Sunagawa et al., 2015). Our data shows that temperature is central for regulating the composition and functioning of cyanobacterial communities. Global warming can affect growth rates and bloom potentials of many taxa within this phylum (Fu et al., 2007; Paerl and Huisman, 2008; Flombaum et al., 2013; Pittera et al., 2014). Niche based models predict an increase in the absolute levels of organisms formerly classified as *Prochlorococcus* and *Synechococcus* due to global warming (Flombaum et al., 2013). Consequently, the functioning of the biogeochemical cycles in which these organisms are involved will also be affected (Fu et al., 2007). Nevertheless, much less is known regarding how global warming could affect communities of Cyanobacteria aside from these two groups of organisms.

The ecogenomic groups identified and their associations with environmental parameters shed light into the potential changes that communities of Cyanobacteria will undergo following global climate changes. Our results indicate that an increase in temperature will lead to decreases in the relative abundances of Low Temperature and Low Temperature Copiotroph groups, while that of High Temperature Oligotroph group increases, especially those of species *Eurycolium neptunis, E. ponticus, E. chisholmi*, and *E. nereus*. One major impact of this alteration is a possible effect on the degree of nitrogen fixation mediated by Cyanobacteria, as none of the species assigned to the High Temperature Oligotroph group are known to fix nitrogen (Latysheva et al., 2012). In fact, our data shows that higher temperatures are associated with lower relative abundances of nitrogen fixating Cyanobacteria of the genera *Trichodesmium* and *Anabaena* (Zehr, 2011). Both beneficial and deleterious effects of the ocean warming and associated phenomena (e.g., acidification) on the rates of growth and N_2_ fixation have been reported (Hutchins et al., 2007; Shi et al., 2012; Fu et al., 2014), and recent laboratory and field experiments (Hong et al., 2017) showed that the acidification inhibit growth and N_2_ fixation in *T. erythraeum* IMS101^T^ due a decrease in cytosolic pH resulting biochemical cost of proton pumping across membranes. Rising temperatures might shift cyanobacterial community composition toward a state were diazotrophs are relatively less abundant. Because nitrogen is often a limiting nutrient to marine primary productivity (Tyrrell, 1999; Moore et al., 2013), alterations in the oceanic levels of nitrogen fixation could affect not only non-diazotrophic Cyanobacteria but also heterotrophic microbes as well as the higher tropic levels that are sustained by microorganisms.

Furthermore, our findings suggest that changes in temperature can affect the contributions of Cyanobacteria to the global carbon pump (Flombaum et al., 2013; Biller et al., 2015). For example, the five strongest positive correlations with temperature between the High Temperature Oligotroph group involve the high-light adapted members of the *Eurycolium* genus (i.e., strains MIT9312^T^, MIT9301^T^, MIT9215, MIT9202^T^, and AS9601^T^). These are high-light adapted strains that display lower photosynthetic efficiency than their low-light adapted counterparts (Moore et al., 1998; Moore and Chisholm, 1999). Our results suggest that the relative abundance of high-light adapted strains would increase induced by the rising temperatures. In turn, these changes could affect the efficiency of carbon fixation in the ocean, a change that could also be influenced by the alterations in nitrogen fixation mentioned above.

## Conclusions

The present study proposes a first attempt toward integrating taxonomy and ecogenomics, offering a compelling new perspective for the development of Cyanobacteria studies. Our results show that closely related genomes often share a niche and can be assigned to the same ecogenomic group. End-users of Cyanobacteria taxonomy may benefit from a more reproducible and portable taxonomic scheme. Future studies are needed to expand the evolutionary and physiological basis for the cyanobacterial niche occupancy, integrating other important ecological variables such as phage susceptibility, light utilization strategies, horizontal gene transfer, and inter-species interactions.

## Author contributions

All authors contributed to the writing of the manuscript. JW, FC, BD, JS, FT, and CT designed and planned the study. JW and FC performed the bioinformatics analyses, analyzed the results, and compiled the data. All authors approved the final version of the manuscript.

### Conflict of interest statement

The authors declare that the research was conducted in the absence of any commercial or financial relationships that could be construed as a potential conflict of interest. The handling Editor declared a shared affiliation, though no other collaboration, with several of the authors [JW, FC, FT, CT].

## Supplementary material

Figure S1 Ribosomal phylogenetic reconstruction of the Cyanobacteria phylum. Tree was constructed through ML using the Kimura 2-parameter method, and GTR+G substitution model. Tree was inferred from 110 16S rRNA gene sequences (~1,400 bp). The species cut-off was 98.8% similarity (Thompson et al., 2015). The percentages of replicate trees in which the associated taxa clustered together in the bootstrap test (1,000 replicates) are shown next to the branches. Nodes supported with a bootstrap of ≥ 50% are indicated. Overwritten T indicates type strain or type species of validly published species to assess their correct phylogenetic assignations. Bold names indicate the additional type strains or type species (only for 16S tree). The unit of measure for the scale bars is the number of nucleotide substitutions per site. *Coleofasciculus chthonoplastes* PCC 7420 is also called *Microcoleus chthonoplastes* PCC 7420. *Gloeobacter violaceus* PCC 7421 sequence was designated as outgroup.

Figure S2 Heatmaps based on GGD metrics of specific cases. **(A)** Heatmap of GGD values between *Oscillatoria* group (case I), where *Microcoleus vaginatus* FGP-2 type strain was included to show the closest relationship with the PCC 7112 strain; **(B)** Heatmap of GGD values between *Leptolyngbya* group (case II); **(C)** Heatmap of GGD values between *Arthrospira* group (case III); and **(D)** Heatmap of GGD values between *Synechococcus* group (case IV). The intraspecies limit is assumed as ≥70% GGD. The GGD values are associated with the respective thermal color scale located at the bottom left corner of the figure. The proposed new names were adopted in this figure.

Figure S3 Abundance and distribution of ecogenomic clusters across global marine metagenomes. Relative abundance of Low Temperature group; Low Temperature Copiotroph group; and High Temperature Oligotroph group at the global scale.

Figure S4 Abundance and distribution of ecogenomic clusters across freshwater metagenomes. **(A)** Relative abundance of ecogenomic clusters in Caatinga biome (metagenomes, *N* = 8). **(B)** Non-metric multidimensional scaling (NMDS) analysis of the freshwater metagenomes and environmental parameters. Ordination plot of physicochemical parameters. Dots indicate the metagenomes samples. Distances were calculated based on the Bray-Curtis Method. NMDS stress value = 0.15. **(C)** Non-metric multidimensional scaling (NMDS) analysis of the freshwater metagenomes and environmental parameters Ordination plot of ecogenomic clusters. Dots indicate the metagenomes samples. Distances were calculated based on the Bray-Curtis Method. NMDS stress value = 0.15.

Table S1 Estimates of genome relatedness of cyanobacterium strains. Values at the matrix indicates the intergenomic distances (i.e., evolutionary divergence between sequences). The numbers of base substitutions per site between sequences are shown. Analyses were conducted accordingly Tamura et al. method. The analysis involved 110 nucleotide sequences. All positions containing gaps and missing data were eliminated. There were a total of 759 positions in the final dataset. Evolutionary analyses were conducted in MEGA6.

Table S2 Details of all cyanobacterial genomes included in this study. Information of other classifications for comparison: NCBI (order and family), numeral identification and genera according to Kozlov et al. (2016), and identification based in curated database CyanoType v.1 (Ramos et al., 2017). Overwritten T indicates type strain or type species.

Data Sheet 1 Formal description of new genera and species.

## References

[B1] Adeolu M. Alnajar S. Naushad S. Gupta R. (2016). Genome-based phylogeny and taxonomy of the ‘Enterobacteriales’: proposal for *Enterobacterales* ord. nov. divided into the families *Enterobacteriaceae, Erwiniaceae* fam. nov., *Pectobacteriaceae* fam. nov., *Yersiniaceae* fam. nov., *Hafniaceae* fam. nov., *Morganellaceae* fam. nov., and *Budviciaceae* fam. nov. Int. J. Syst. Evol. Microbiol. 66, 5575–5599. 10.1099/ijsem.0.001485 27620848

[B2] Ahn A. C. Meier-Kolthoff J. P. Overmars L. Richter M. Woyke T. Sorokin D. Y.. (2017). Genomic diversity within the haloalkaliphilic genus *Thioalkalivibrio*. PLoS ONE 12:e0173517. 10.1371/journal.pone.0173517 28282461 PMC5345834

[B3] Almstrand R. Pinto A. J. Figueroa L. A. Sharp J. O. (2016). Draft genome sequence of a novel *Desulfobacteraceae* member from a sulfate-reducing bioreactor metagenome. Genome Announc. 4:e01540-15. 10.1128/genomeA.01540-15 26769931 PMC4714113

[B4] Al-saari N. Gao F. Rohul A. A. K. M. Sato K. Sato K. Mino S.. (2015). Advanced microbial taxonomy combined with genome-based-approaches reveals that *Vibrio astriarenae* sp. nov., an agarolytic marine bacterium, forms a new clade in *vibrionaceae*. PLoS ONE 10:e0136279. 10.1371/journal.pone.0136279 26313925 PMC4551953

[B5] Amin A. K. M. R. Tanaka M. Al-Saari N. Feng G. Mino S. Ogura Y.. (2017). *Thaumasiovibrio occultus gen*. nov. sp. nov. and *Thaumasiovibrio subtropicus* sp. nov. within the family *Vibrionaceae*, isolated from coral reef seawater off Ishigaki Island, Japan. Syst. Appl. Microbiol. 40, 290–296. 10.1016/j.syapm.2017.04.003 28648725

[B6] Anantharaman K. Brown C. T. Hug L. A. Sharon I. Castelle C. J. Probst A. J.. (2016). Thousands of microbial genomes shed light on interconnected biogeochemical processes in an aquifer system. Nat. Commun. 7:13219. 10.1038/ncomms13219 27774985 PMC5079060

[B7] Appolinario L. R. Tschoeke D. A. Rua C. P. Venas J. T. Campeão M. E. Amaral G. R. S. (2016). Description of *Endozoicomonas arenosclerae* sp. nov. using a genomic taxonomy approach. Anton. Van Leeuwenhoek. 109, 431–438. 10.1007/s10482-016-0649-x 26786501

[B8] Auch A. F. Klenk H.-P. Göker M. (2010b). Standard operating procedure for calculating genome-to-genome distances based on high-scoring segment pairs. Stand. Genomic Sci. 2, 142–148. 10.4056/sigs.541628 21304686 PMC3035261

[B9] Auch A. F. von Jan M. Klenk H.-P. Göker M. (2010a). Digital DNA-DNA hybridization for microbial species delineation by means of genome-to-genome sequence comparison. Stand. Genomic Sci. 2, 117–134. 10.4056/sigs.531120 21304684 PMC3035253

[B10] Batut B. Knibbe C. Marais G. Daubin V. (2014). Reductive genome evolution at both ends of the bacterial population size spectrum. Nat. Rev. Microb. 12, 841–850. 10.1038/nrmicro3331 25220308

[B11] Becraft E. D. Wood J. M. Rusch D. B. Kühl M. Jensen S. I. Bryant D. A.. (2015). The molecular dimension of microbial species: 1. ecological distinctions among, and homogeneity within, putative ecotypes of *Synechococcus* inhabiting the cyanobacterial mat of Mushroom Spring, Yellowstone National Park. Front. Microbiol. 6:590. 10.3389/fmicb.2015.00590 26157420 PMC4475828

[B12] Beiko R. G. (2015). Microbial malaise: how can we classify the microbiome? Trends Microbiol. 23, 671–679. 10.1016/j.tim.2015.08.009 26439295

[B13] Biller S. J. Berube P. M. Lindell D. Chisholm S. W. (2015). *Prochlorococcus*: the structure and function of collective diversity. Nat. Rev. Microb. 13, 13–27. 10.1038/nrmicro3378 25435307

[B14] Boone D. R. Castenholz R. W. (2001). Bergey's manual of systematic bacteriology volume one, in The Archaea and the Deeply Branching and Phototrophic Bacteria, 2nd Edn., eds Garrity G. Boone D. R. Castenholz R. W. (New York, NY: Springer-Verlag), 473–487.

[B15] Brown C. T. Hug L. A. Thomas B. C. Sharon I. Castelle C. J. Singh A.. (2015). Unusual biology across a group comprising more than 15% of domain bacteria. Nature 523, 208–211. 10.1038/nature14486 26083755

[B16] Castresana J. (2000). Selection of conserved blocks from multiple alignments for their use in phylogenetic analysis. Mol. Biol. Evol. 17, 540–552. 10.1093/oxfordjournals.molbev.a026334 10742046

[B17] Choudoir M. L. Campbell A. N. Buckley D. H. (2012). Grappling with Proteus: population-level approaches to understanding microbial diversity. Front. Microb. 3:336. 10.3389/fmicb.2012.00336 23024645 PMC3441200

[B18] Chun J. Rainey F. A. (2014). Integrating genomics into the taxonomy and systematics of the Bacteria and Archaea. Int. J. Syst. Evol. Microbiol. 64, 316–324. 10.1099/ijs.0.054171-0 24505069

[B19] Coenye T. Gevers D. Van de Peer Y. Vandamme P. Swings J. (2005). Towards a prokaryotic genomic taxonomy. FEMS Microbiol. Rev. 29, 147–167. 10.1016/j.femsre.2004.11.004 15808739

[B20] Coutinho F. Dutilh B. E. Thompson C. Thompson F. (2016b). Proposal of fifteen new species of *Parasynechococcus* based on genomic, physiological and ecological features. Arch. Microbiol. 198, 973–986. 10.1007/s00203-016-1256-y 27339259

[B21] Coutinho F. Tschoeke D. A. Thompson F. Thompson C. (2016a). Comparative genomics of *Synechococcus* and proposal of the new genus Parasynechococcus. Peer J. 4:e1522. 10.7717/peerj.1522 26839740 PMC4734447

[B22] De Vos P. Trüper H. G. (2000). Judicial Commission of the International Committee on systematic bacteriology. Int. J. Syst. Evol. Microbiol. 50, 2239–2244. 10.1099/00207713-50-6-2239

[B23] Di Rienzi S. C. Sharon I. Wrighton K. C. Koren O. Hug L. A. Thomas B. C.. (2013). The human gut and groundwater harbor non-photosynthetic bacteria belonging to a new candidate phylum sibling to *Cyanobacteria*. Elife 2:e01102. 10.7554/eLife.01102 24137540 PMC3787301

[B24] Edgar R. C. (2004). MUSCLE: multiple sequence alignment with high accuracy and high throughput. Nucleic Acids Res. 32, 1792–1797. 10.1093/nar/gkh340 15034147 PMC390337

[B25] Farrant G. K. Doré H. Cornejo-Castillo F. M. Partensky F. Ratin M. Ostrowski M.. (2016). Delineating ecologically significant taxonomic units from global patterns of marine picocyanobacteria. Proc. Natl. Acad. Sci. U.S.A. 113, E3365–E3374. 10.1073/pnas.1524865113 27302952 PMC4914166

[B26] Flombaum P. Gallegos J. L. Gordillo R. A. Rincón J. Zabala L. L. Jiao N.. (2013). Present and future global distributions of the marine cyanobacteria *Prochlorococcus* and *Synechococcus*. Proc. Natl. Acad. Sci. U.S.A. 110, 9824–9829. 10.1073/pnas.1307701110 23703908 PMC3683724

[B27] Fu F.-X. Warner M. E. Zhang Y. Feng Y. Hutchins D. A. (2007). Effects of increased temperature and CO_2_ on photosynthesis, growth, and elemental ratios in marine *Synechococcus* and *Prochlorococcus*. J. Phycol. 43, 485–496. 10.1111/j.1529-8817.2007.00355.x

[B28] Fu F.-X. Yu E. Garcia N. S. Gale J. Luo Y. Webb E. A. (2014). Differing responses of marine N2 fixers to warming and consequences for future diazotroph community structure. Aquat. Microb. Ecol. 72, 33–46. 10.3354/ame01683

[B29] Garrity G. M. (2016). A new genomics-driven taxonomy of Bacteria and Archaea: are we there yet? J. Clin. Microb. 54, 1956–1963. 10.1128/JCM.00200-16 27194687 PMC4963521

[B30] Garrity G. M. Oren A. (2012). Response to Gribaldo and Brochier-Armanet: time for order in microbial systematics. Trends Microbiol. 20, 353–354. 10.1016/j.tim.2012.05.004 22704611

[B31] Gevers D. Cohan F. M. Lawrence J. G. Spratt B. G. Coenye T. Feil E. J.. (2005). Re-evaluating prokaryotic species. Nat. Rev. Microbiol. 3, 733–739. 10.1038/nrmicro1236 16138101

[B32] Goris J. Konstantinidis K. T. Klappenbach J. A. Coenye T. Vandamme P. Tiedje J. M. (2007). DNA-DNA hybridization values and their relationship to whole-genome sequence similarities. Int. J. Syst. Evol. Microbiol. 14, 81–91. 10.1099/ijs.0.64483-0 17220447

[B33] Gribaldo S. Brochier-Armanet C. (2012). Time for order in microbial systematics. Trends Microbiol. 20, 209–210. 10.1016/j.tim.2012.02.006 22440793

[B34] Gugger M. F. Hoffmann L. (2004). Polyphyly of true branching *cyanobacteria* (Stigonematales). Int. J. Syst. Evol. Microbiol. 54, 349–357. 10.1099/ijs.0.02744-0 15023942

[B35] Gupta R. S. Naushad S. Baker S. (2015). Phylogenomic analyses and molecular signatures for the class *Halobacteria* and its two major clades: a proposal for division of the class *Halobacteria* into an emended order *Halobacteriales* and two new orders, *Haloferacales* ord. nov. and *Natrialbales* ord. nov., containing the novel families *Haloferacaceae* fam. nov. and *Natrialbaceae* fam. nov. Int. J. Syst. Evol. Microbiol. 65, 1050–1069. 10.1099/ijs.0.070136-0 25428416

[B36] Hahnke R. L. Meier-Kolthoff J. P. García-López M. Mukherjee S. Huntemann M. Ivanova N. N.. (2016). Genome-based taxonomic classification of bacteroidetes. Front. Microbiol. 7:2003. 10.3389/fmicb.2016.02003 28066339 PMC5167729

[B37] Haroon M. F. Thompson L. R. Parks D. H. Hugenholstz P. Stingl U. (2016). Data descriptor: a catalogue of 136 microbial draft genomes from Red Sea metagenomes. Sci. Data 3:160050 10.1038/sdata.2016.50 27377622 PMC4932879

[B38] Hoffmann L. Komárek J. Kaštovský J. (2005). System of cyanoprokaryotes (cyanobacteria) – state in 2004. Arch. Hydrobiol. Suppl. Algol. Stud. 117, 95–115. 10.1127/1864-1318/2005/0117-0095

[B39] Hong H. Shen R. Zhang F. Wen Z. Chang S. Lin W.. (2017). The complex effects of ocean acidification on the prominent N2-fixing cyanobacterium *Trichodesmium*. Science 356, 527–531. 10.1126/science.aal2981 28450383

[B40] Hug L. A. Baker B. J. Anantharaman K. Brown C. T. Probst A. J. Castelle C. J. (2016). A new view of the tree and life. Nat. Microbiol. 1:16048 10.1038/nmicrobiol.2016.48 27572647

[B41] Hugenholtz P. Skarshewski A. Parks D. H. (2016). Genome-based microbial taxonomy coming of age. Cold Spring Harb. Perspect. Biol. 8:a018085. 10.1101/cshperspect.a018085 26988968 PMC4888819

[B42] Hugerth L. W. Larsson J. Alneberg J. Lingh M. Legrand C. Pinhassi J.. (2015). Metagenome-assembled genomes uncover a global brackish microbiome. Genome Biol. 16:279. 10.1186/s13059-015-0834-7 26667648 PMC4699468

[B43] Hutchins D. A. Fu F. X. Zhang Y. Warner M. E. Feng Y. Portune K. (2007). CO_2_ control of *Trichodesmium* N2 fixation, photosynthesis, growth rates, and elementalratios: implicationsfor past, present, and future ocean biogeochemistry. Limnol. Oceanogr. 52, 1293–1304. 10.4319/lo.2007.52.4.1293

[B44] Iverson V. Morris R. M. Frazar C. D. Berthiaume C. T. Morales R. L. Armbrust E. V. (2012). Untangling genomes from metagenomes: revealing an uncultured class of marine Euryarchaeota. Science 335, 587–590. 10.1126/science.1212665 22301318

[B45] Kauff F. Büdel B. (2010). Phylogeny of cyanobacteria: an overview. Prog. Bot. 72, 209–224. 10.1007/978-3-642-13145-5_8

[B46] Komárek J. Kastovsky J. Mares J. Johansen J. R. (2014). Taxonomic classification of cyanoprokaryotes (cyanobacterial genera) 2014, using a polyphasic approach. Preslia 86, 295–335.

[B47] Konstantinidis K. T. Ramette A. Tiedje J. M. (2006). The bacterial species definition in the genomic era. Philos. Trans. R. Soc. B. 361, 1929–1940. 10.1098/rstb.2006.1920 17062412 PMC1764935

[B48] Konstantinidis K. T. Tiedje J. M. (2005a). Genomic insights that advance the species definition for prokaryotes. Proc. Natl. Acad. Sci. U.S.A. 102, 2567–2572. 10.1073/pnas.0409727102 15701695 PMC549018

[B49] Konstantinidis K. T. Tiedje J. M. (2005b). Towards a genome-based taxonomy for prokaryotes. J. Bacteriol. 187, 6258–6264. 10.1128/JB.187.18.6258-6264.2005 16159757 PMC1236649

[B50] Kozlov A. M. Zhang J. Yilmaz P. Glöckner F. O. Stamatakis A. (2016). Phylogeny-aware identification and correction of taxonomically mislabeled sequences. Nucleic Acids Res. 44, 5022–5033. 10.1093/nar/gkw396 27166378 PMC4914121

[B51] Labeda D. P. (2000). International Committee on Systematic Bacteriology IXth International (IUMS) congress of bacteriology and applied. Int. J. Syst. Evol. Microbiol. 50, 2245–2247. 10.1099/00207713-50-6-2245

[B52] Lagesen K. Hallin P. Rodland M. E. A. Staerfeldt H.-H. Rognes T. Ussery D. (2007). RNAmmer: consistent and rapid annotation of ribosomal RNA genes. Nucleic Acids Res. 35, 3100–3108. 10.1093/nar/gkm160 17452365 PMC1888812

[B53] Langmead B. Salzberg S. L. (2012). Fast gapped-read alignment with Bowtie 2. Nat. Methods 9, 357–359. 10.1038/nmeth.1923 22388286 PMC3322381

[B54] Latysheva N. Junker V. L. Palmer W. J. Codd G. A. Barker D. (2012). The evolution of nitrogen fixation in cyanobacteria. Bioinformatics 28, 603–606. 10.1093/bioinformatics/bts008 22238262

[B55] Locey K. J. Lennon J. T. (2016). Scaling laws predict global microbial diversity. Proc. Natl. Acad. Sci. U.S.A. 113, 5970–5975. 10.1073/pnas.1521291113 27140646 PMC4889364

[B56] Lopes F. A. C. Catão E. C. P. Santana R. H. Cabral A. S. Paranhos R. Rangel T. P.. (2016). Microbial community profile and water quality in a protected area of the Caatinga biome. PLoS ONE 11:e0148296. 10.1371/journal.pone.0148296 26881432 PMC4755664

[B57] Meier-Kolthoff J. P. Auch A. F. Klenk H.-P. Göker M. (2013). Genome sequence-based species delimitation with confidence intervals and improved distance functions. BMC Bioinformatics 14:60. 10.1186/1471-2105-14-60 23432962 PMC3665452

[B58] Moore C. M. Mills M. M. Arrigo K. R. Berman-Frank I. Bopp L. Boyd P. W. (2013). Processes and patterns of oceanic nutrient limitation. Nat. Geosci. 6, 701–710. 10.1038/ngeo1765

[B59] Moore L. R. Chisholm S. W. (1999). Photophysiology of the marine cyanobacterium *Prochlorococcus*: ecotypic differences among cultured isolates. Limnol. Oceanogr. 44, 628–638.

[B60] Moore L. R. Rocap G. Chisholm S. W. (1998). Physiology and molecular phylogeny of coexisting *Prochlorococcus* ecotypes. Nature 393, 464–467. 9624000 10.1038/30965

[B61] Oren A. (2004). A proposal for further integration of the cyanobacteria under the bacteriological code. Int. J. Syst. Evol. Microbiol. 54, 1895–1902. 10.1099/ijs.0.03008-0 15388760

[B62] Oren A. Garrity G. M. (2014). Proposal to change general consideration 5 and principle 2 of the international code of nomenclature of prokaryotes. Int. J. Syst. Evol. Microbiol. 64, 309–310. 10.1099/ijs.0.059568-0 24408952

[B63] Oren A. Komárek J. Hoffmann L. (2009). Nomenclature of the Cyanophyta/Cyanobacteria/Cyanoprokaryotes – What has happened since IAC Luxembourg? Arch. Hydrobiol. Suppl. Algol. Stud. 130, 17–26. 10.1127/1864-1318/2009/0130-0017

[B64] Oren A. Tindall B. J. (2005). Nomenclature of the cyanophyta/cyanobacteria/cyanoprokaryotes under the international code of nomenclature of prokaryotes. Arch. Hydrobiol. Suppl. Algol. Stud. 117, 39–52. 10.1127/1864-1318/2005/0117-0039

[B65] Oren A. Ventura S. (2017). The current status of cyanobacterial nomenclature under the “prokaryotic” and the “botanical” code. Antonie van Leeuwenhoek. 110, 1257–1269. 10.1007/s10482-017-0848-0 28243951

[B66] Paerl H. W. Huisman J. (2008). Blooms like it hot. Science 320, 57–58. 10.1126/science.1155398 18388279

[B67] Parks D. H. Imelfort M. Skennerton C. T. Hugenholtz P. Tyson G. W. (2015). CheckM: assessing the quality of microbial genomes recovered from isolates, single cells, and metagenomes. Genome Res. 25, 1043–1055. 10.1101/gr.186072.114 25977477 PMC4484387

[B68] Parte A. C. (2014). LPSN - List of prokaryotic names with standing in nomenclature. Nucleic Acids Res. 42, 613–616. 10.1093/nar/gkt1111 24243842 PMC3965054

[B69] Pinto A. J. Sharp J. O. Yoder M. J. Almstrand R. (2016). Draft genome sequences of two novel Acidimicrobiaceae members from an acid mine drainage biofilm metagenome. Genome Announc. 4:e01563-15. 10.1128/genomeA.01563-15 26769942 PMC4714123

[B70] Pittera J. Humily F. Thorel M. Grulois D. Garczarek L. Six C. (2014). Connecting thermal physiology and latitudinal niche partitioning in marine Synechococcus. ISME J. 8, 1221–1236. 10.1038/ismej.2013.228 24401861 PMC4030225

[B71] Pruesse E. Quast C. Knittel K. Fuchs B. M. Ludwig W. Peplies J.. (2007). SILVA: a comprehensive online resource for quality checked and aligned ribosomal RNA sequence data compatible with ARB. Nucleic Acids Res. 35, 7188–7196. 10.1093/nar/gkm864 17947321 PMC2175337

[B72] Puigbò P. Lobkovsky A. E. Kristensen D. M. Wolf Y. I. Koonin E. V. (2014). Genomes in turmoil: quantification of genome dynamics in prokaryote supergenomes. BMC Biol. 12:66. 10.1186/s12915-014-0066-4 25141959 PMC4166000

[B73] Qin Q. L. Xie B.-B. Zhang X.-Y. Chen X.-L. Zhou B.-C. Zhou J.. (2014). A proposed genus boundary for the prokaryotes based on genomic insights. J. Bacteriol. 196, 2210–2215. 10.1128/JB.01688-14 24706738 PMC4054180

[B74] Quast C. Pruesse E. Yilmaz P. Gerken J. Schweer T. Yarza P.. (2013). The SILVA ribosomal RNA gene database project: improved data processing and web-based tools. Nucleic Acids Res. 41, D590–D596. 10.1093/nar/gks1219 23193283 PMC3531112

[B76] R Development Core Team (2011). R: A Language and Environment for Statistical Computing. Vienna: The R Foundation for Statistical Computing Available online at: http://www.R-project.org/

[B75] Rajaniemi R. Hrouzek P. Kastovska K. Willame R. Rantala A. Hoffmann L.. (2005). Phylogenetic and morphological evaluation of the genera Anabaena, Aphanizomenon, Trichormus and Nostoc (Nostocales, Cyanobacteria). Int. J. Syst. Evol. Microbiol. 55, 11–26. 10.1099/ijs.0.63276-0 15653847

[B77] Rambaut A. (2015). FigTree v1.4.2: Tree figure drawing tool. Available online at: http://tree.bio.ed.ac.uk/software/figtree/

[B78] Ramos V. Morais J. Vasconcelos V. M. (2017). A curated database of cyanobacterial strains relevant for modern taxonomy and phylogenetic studies. Sci. Data 4:170054. 10.1038/sdata.2017.54 28440791 PMC5404626

[B79] Richter M. Rossello-Mora R. (2009). Shifting the genomic gold standard for the prokaryotic species definition. Proc. Natl. Acad. Sci. U.S.A. 106, 19126–19131. 10.1073/pnas.0906412106 19855009 PMC2776425

[B80] Rippka R. Deruelles J. Waterbury J. B. Herdman M. Stanier R. Y. (1979). Generic assignments, strain histories and properties of pure cultures of *Cyanobacteria*. J. Gen. Microb. 111, 1–61.

[B81] Rossello-Mora R. Amann R. (2001). The species concept for prokaryotes. FEMS Microbiol. Rev. 25, 39–67. 10.1111/j.1574-6976.2001.tb00571.x 11152940

[B82] Sarma T. A. (2012). Handbook of Cyanobacteria. Boca Raton, FL: CRC Press.

[B83] Schirrmeister B. E. Anisimova M. Antonelli A. Bagheri H. C. (2011b). Evolution of cyanobacterial morphotypes: taxa required for improved phylogenomic approaches. Commun. Integr. Biol. 4, 424–427. 10.4161/cib.4.4.16183 21966561 PMC3181511

[B84] Schirrmeister B. E. Antonelli A. Bagheri H. C. (2011a). The origin of multicellularity in cyanobacteria. Evol. Biol. 11:45. 10.1186/1471-2148-11-45 21320320 PMC3271361

[B85] Seemann T. (2014). Prokka: rapid prokaryotic genome annotation. Bioinformatics 30, 2068–2069. 10.1093/bioinformatics/btu153 24642063

[B86] Shi D. Kranz S. A. Kim J.-M. Morel F. M. M. (2012). Ocean acidification slows nitrogen fixation and growth in the dominant diazotroph *Trichodesmium* under low-iron conditions. Proc. Natl. Acad. Sci. U.S.A. 109, E3094–E3100. 10.1073/pnas.1216012109 23071328 PMC3494951

[B87] Shih P. M. Wu D. Latifi A. Axen S. D. Fewer D. P. Talla E.. (2013). Improving the coverage of the cyanobacterial phylum using diversity-driven genome sequencing. Proc. Natl. Acad. Sci. U.S.A. 110, 1053–1058. 10.1073/pnas.1217107110 23277585 PMC3549136

[B88] Soo R. M. Skennerton C. T. Sekiguchi Y. Imelfort M. Paech S. J. Dennis P. G.. (2014). An expanded genomic representation of the phylum *Cyanobacteria*. Genome Biol. Evol. 6, 1031–1045. 10.1093/gbe/evu073 24709563 PMC4040986

[B89] Soo R. M. Woodcroft B. J. Parks D. H. Tyson G. W. Hugenholtz P. (2015). Back from the dead; the curious tale of the predatory cyanobacterium *Vampirovibrio chlorellavorus*. Peer J. 3:e968. 10.7717/peerj.968 26038723 PMC4451040

[B90] Spang A. Saw J. H. Jorgensen S. L. Zaremba-Niedzwiedzka K. Martijn J. Lind A. E.. (2015). Complex archaea that bridge the gap between prokaryotes and eukaryotes. Nature 521, 173–179. 10.1038/nature14447 25945739 PMC4444528

[B91] Stackenbrandt E. Frederiksen W. Garrity G. M. Grimont P. A. Kampfer P. Maiden M. C. (2002). Report of the *ad hoc* committee for the re-evaluation of the species definition in bacteriology. Int. J. Syst. Evol. Microbiol. 52, 1043–1047. 10.1099/00207713-52-3-1043 12054223

[B92] Stamatakis A. (2006). RAxML-VI-HPC: maximum likelihood-based phylogenetic analyses with thousands of taxa and mixed models. Bioinformatics 22, 2688–2690. 10.1093/bioinformatics/btl446 16928733

[B93] Stanier R. Y. Sistrom W. R. Hansen T. A. Whitton B. A. Castenholz R. W. Pfennig N. (1978). Proposal to place the nomenclature of the *Cyanobacteria* (Blue-Green Algae) under the rules of the international code of nomenclature of bacteria. Int. J. Syst. Bacteriol. 28, 335–336.

[B94] Stocker R. Seymour J. R. (2012). Ecology and physics of bacterial chemotaxis in the ocean. Microbiol. Mol. Biol. Rev. 76, 792–812. 10.1128/MMBR.00029-12 23204367 PMC3510523

[B95] Sunagawa S. Coelho L. P. Chaffron S. Kultima J. R. Labadie K. Salazar G.. (2015). Structure and function of the global ocean microbiome. Science 348:1261359. 10.1126/science.1261359 25999513

[B96] Sutcliffe I. C. Trujillo M. E. Whitman W. B. Goodfellow M. (2013). A call to action for the International Committee on Systematics of Prokaryotes. Trends Microbiol. 21, 51–52. 10.1016/j.tim.2012.11.004 23238020

[B97] Tajima F. Nei M. (1984). Estimation of evolutionary distance between nucleotide sequences. Mol. Biol. Evol. 1, 269–285. 6599968 10.1093/oxfordjournals.molbev.a040317

[B98] Talavera G. Castresana J. (2007). Improvement of phylogenies after removing divergent and ambiguously aligned blocks from protein sequence alignments. Syst. Biol. 56, 564–577. 10.1080/10635150701472164 17654362

[B99] Tamura K. Stecher G. Peterson D. Filipski A. Kumar S. (2013). MEGA6: molecular evolutionary genetics analysis version 6.0. Mol. Biol. Evol. 30, 2725–2729. 10.1093/molbev/mst197 24132122 PMC3840312

[B100] Thompson C. C. Amaral G. R. Campeão M. Edwards R. A. Polz M. F. Dutilh B. E.. (2015). Microbial taxonomy in the post-genomic era: rebuilding from scratch? Arch. Microbiol. 197, 359–370. 10.1007/s00203-014-1071-2 25533848

[B101] Thompson C. C. Chimetto L. Edwards R. A. Swings J. Stackebrandt E. Thompson F. L. (2013b). Microbial genomic taxonomy. BMC Genomics 14:913. 10.1186/1471-2164-14-913 24365132 PMC3879651

[B102] Thompson C. C. Silva G. G. Vieira N. M. Edwards R. Vicente A. C. Thompson F. L. (2013a). Genomic taxonomy of the genus *Prochlorococcus*. Microbial. Ecol. 66, 752–762. 10.1007/s00248-013-0270-8 23963220

[B103] Tindall B. J. (1999). Proposals to update and make changes to the Bacteriological Code. Int. J. Syst. Evol. Microbiol. 49, 1309–1312. 10425795 10.1099/00207713-49-3-1309

[B104] Ting C. S. Rocap G. King J. Chisholm S. W. (2002). Cyanobacterial photosynthesis in the oceans: the origins and significance of divergent light-harvesting strategies. Trends Microbiol. 10, 134–142. 10.1016/S0966-842X(02)02319-3 11864823

[B105] Tyrrell T. (1999). The relative influences of nitrogen and phosphorus on oceanic primary production. Nature 400, 525–531.

[B106] Varghese N. J. Mukherjee S. Ivanova N. Konstantinidis K. T. Mavrommatis K. Kyrpides N. C.. (2015). Microbial species delineation using whole genome sequences. Nucleic Acids Res. 43, 6761–6771. 10.1093/nar/gkv657 26150420 PMC4538840

[B107] Waite D. W. Vanwonterghem I. Rinke C. Parks D. H. Zhang Y. Takai K. (2017). Comparative genomic analysis of the class *Epsilonproteobacteria* and proposed reclassification to epsilonbacteraeota (phyl. Nov.). Front. Microbiol. 24:682 10.3389/fmicb.2017.00682 PMC5401914 28484436

[B108] Wayne L. G. Brenner D. J. Colwell R. R. Grimont P. A. D. Kandler O. Krichevsky M. I. (1987). International Committee on Systematic Bacteriology. Report of the *ad hoc* committee on reconciliation of approaches to bacterial systematics. Int. J. Syst. Bacteriol. 37, 463–464.

[B109] Wu M. Eisen J. (2008). A simple, fast, and accurate method of phylogenomic inference. Genome Biol. 9:R151. 10.1186/gb-2008-9-10-r151 18851752 PMC2760878

[B110] Wu M. Scott A. J. (2012). Phylogenomic analysis of bacterial and archaeal sequences with AMPHORA2. Bioinformatics 28, 1033–1034. 10.1093/bioinformatics/bts079 22332237

[B111] Yarza P. Richter M. Peplies J. Euzeby J. Amann R. Schleifer K.-H.. (2008). The all-species living tree project: a 16S rRNA-based phylogenetic tree of all sequenced type strains. Syst. Appl. Microbiol. 31, 241–250. 10.1016/j.syapm.2008.07.001 18692976

[B112] Yeates T. O. Kerfeld C. A. Heinhorst S. Cannon G. C. Shively J. M. (2008). Protein-based organelles in bacteria: carboxysomes and related microcompartments. Nat. Rev. Microbiol. 6, 681–691. 10.1038/nrmicro1913 18679172

[B113] Zehr J. P. (2011). Nitrogen fixation by marine cyanobacteria. Trends Microbiol. 19, 162–173. 10.1016/j.tim.2010.12.004 21227699

