# Supplementary figures and images for: Ecogenomics and Taxonomy of Cyanobacteria Phylum
Source: Front Microbiol. 2017 Nov 14;8:2132. doi: 10.3389/fmicb.2017.02132 (PMC5694629; doi:10.3389/fmicb.2017.02132)

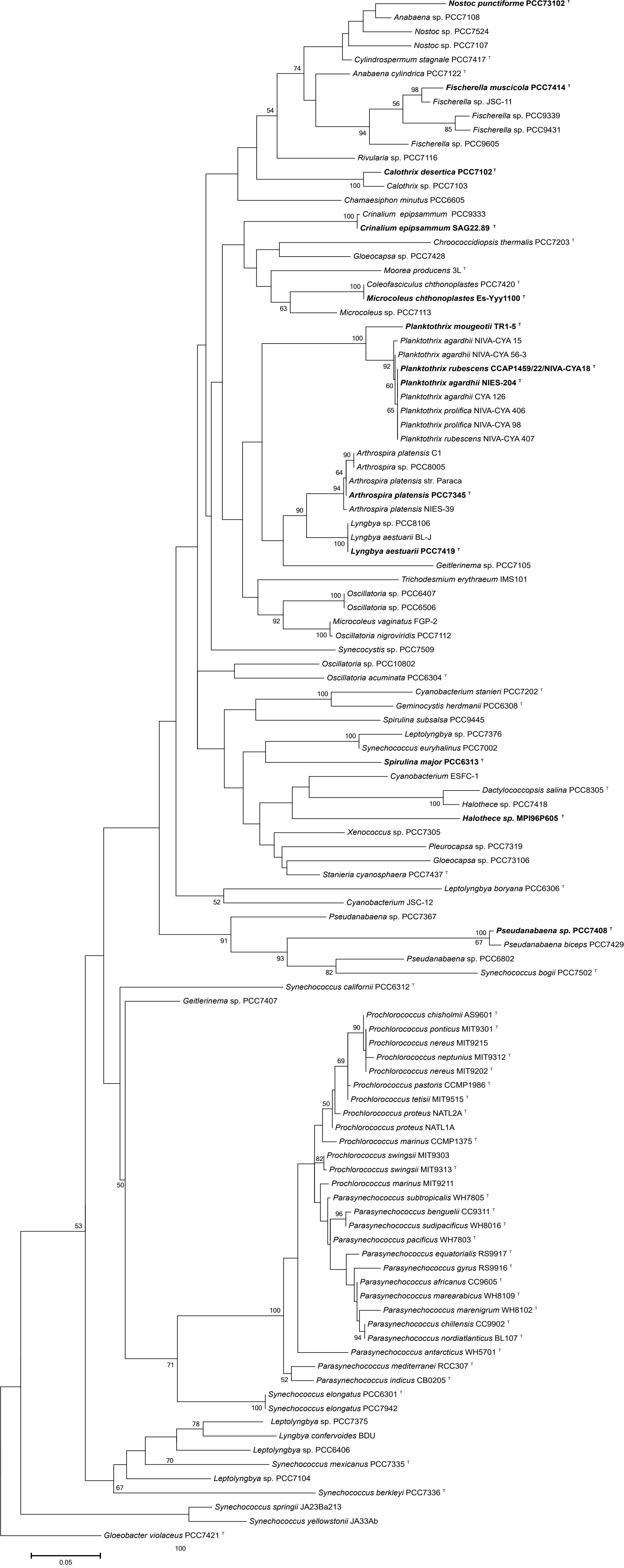

0.05

100

Supplement: Figure S1 — Ribosomal phylogenetic reconstruction of the Cyanobacteria phylum. Tree was constructed through ML using the Kimura 2-parameter method, and GTR+G substitution model. Tree was inferred from 110 16S rRNA gene sequences (~1,400 bp). The species cut-off was 98.8% similarity (Thompson et al., 2015). The percentages of replicate trees in which the associated taxa clustered together in the bootstrap test (1,000 replicates) are shown next to the branches. Nodes supported with a bootstrap of ≥ 50% are indicated. Overwritten T indicates type strain or type species of validly published species to assess their correct phylogenetic assignations. Bold names indicate the additional type strains or type species (only for 16S tree). The unit of measure for the scale bars is the number of nucleotide substitutions per site. Coleofasciculus chthonoplastes PCC 7420 is also called Microcoleus chthonoplastes PCC 7420. Gloeobacter violaceus PCC 7421 sequence was designated as outgroup. [file Image1.PDF]

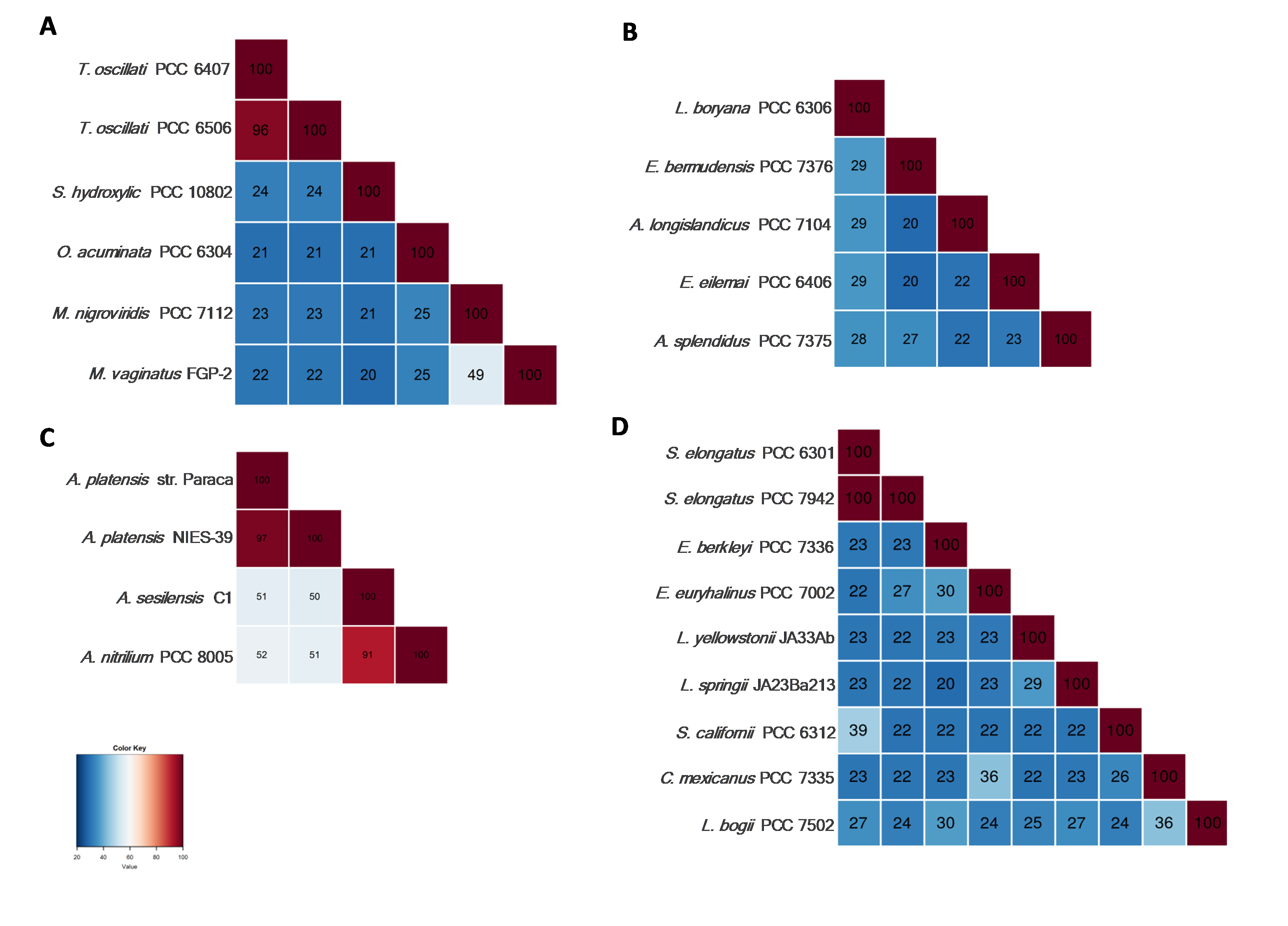

Supplement: Figure S2 — Heatmaps based on GGD metrics of specific cases. (A) Heatmap of GGD values between Oscillatoria group (case I), where Microcoleus vaginatus FGP-2 type strain was included to show the closest relationship with the PCC 7112 strain; (B) Heatmap of GGD values between Leptolyngbya group (case II); (C) Heatmap of GGD values between Arthrospira group (case III); and (D) Heatmap of GGD values between Synechococcus group (case IV). The intraspecies limit is assumed as ≥70% GGD. The GGD values are associated with the respective thermal color scale located at the bottom left corner of the figure. The proposed new names were adopted in this figure. [file Image2.TIF]

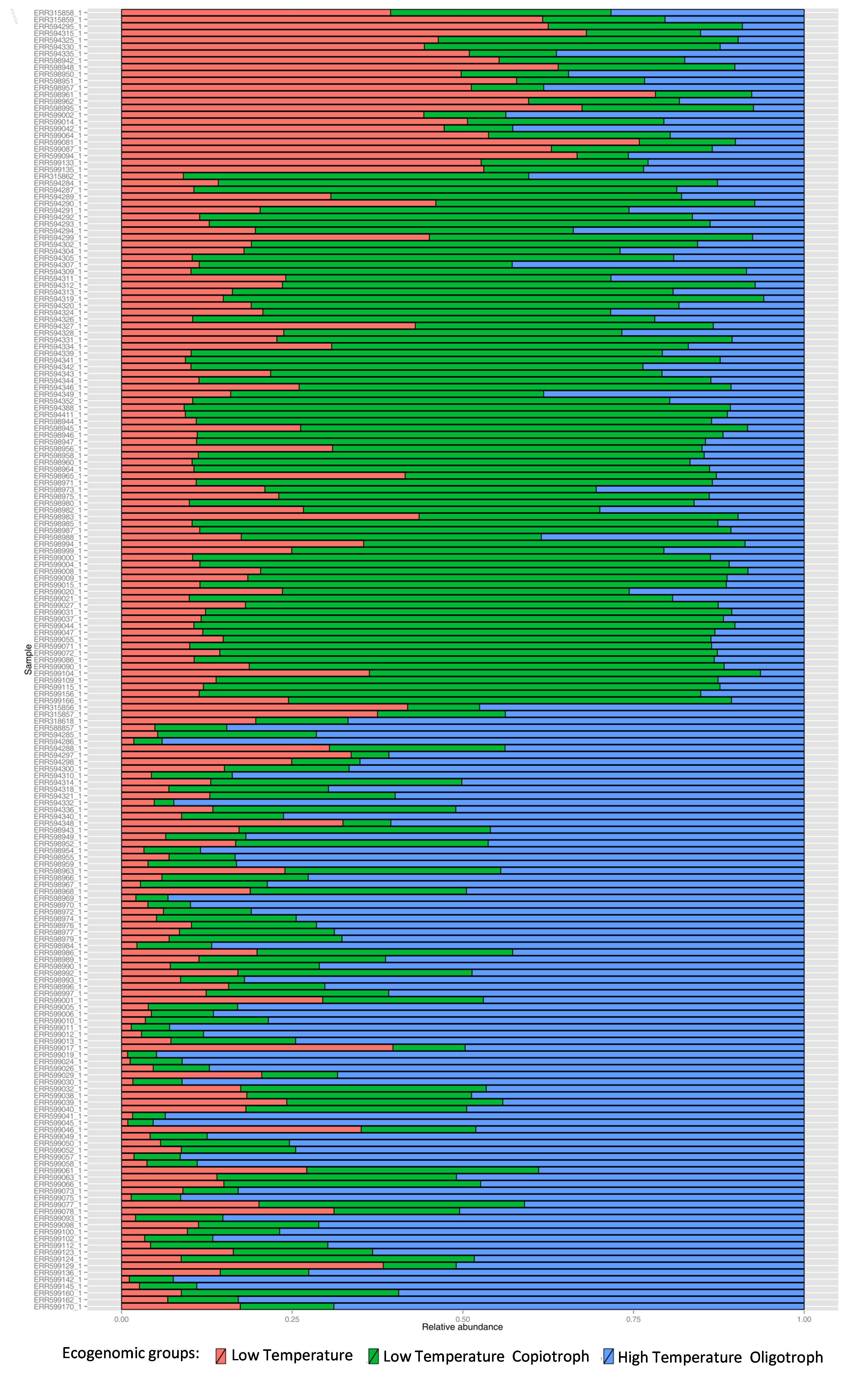

Supplement: Figure S3 — Abundance and distribution of ecogenomic clusters across global marine metagenomes. Relative abundance of Low Temperature group; Low Temperature Copiotroph group; and High Temperature Oligotroph group at the global scale. [file Image3.TIF]

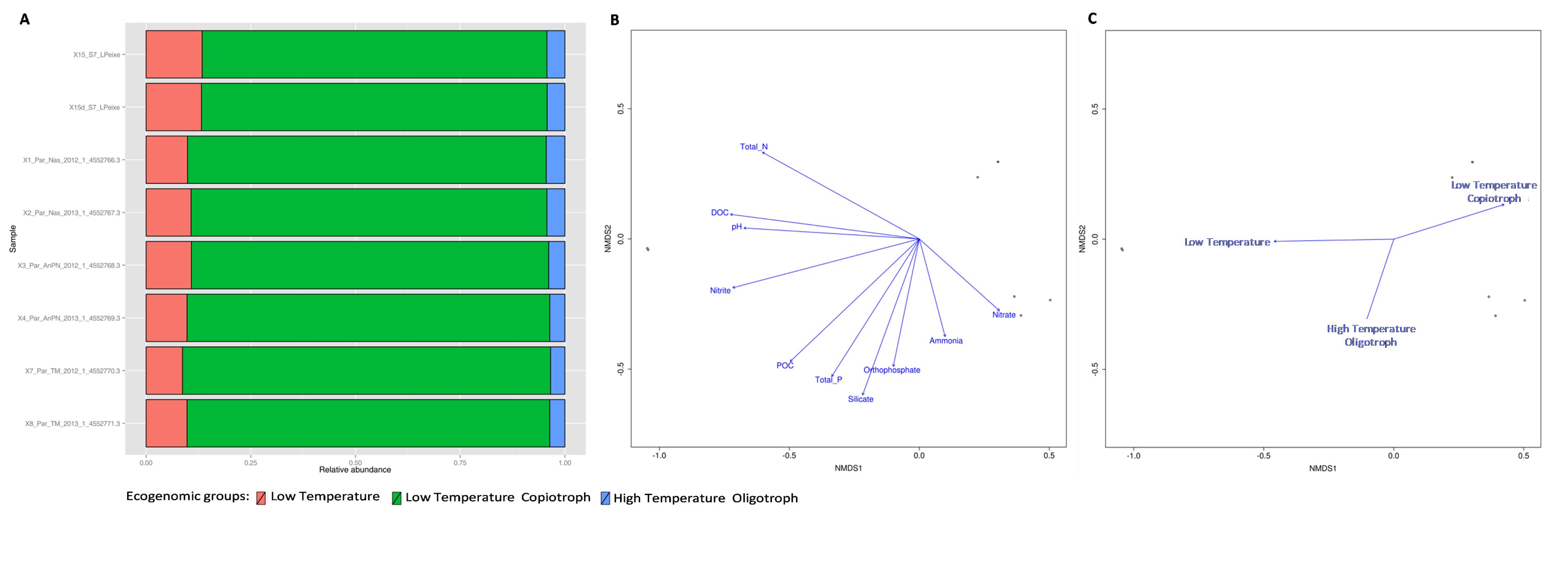

Supplement: Figure S4 — Abundance and distribution of ecogenomic clusters across freshwater metagenomes. (A) Relative abundance of ecogenomic clusters in Caatinga biome (metagenomes, N = 8). (B) Non-metric multidimensional scaling (NMDS) analysis of the freshwater metagenomes and environmental parameters. Ordination plot of physicochemical parameters. Dots indicate the metagenomes samples. Distances were calculated based on the Bray-Curtis Method. NMDS stress value = 0.15. (C) Non-metric multidimensional scaling (NMDS) analysis of the freshwater metagenomes and environmental parameters Ordination plot of ecogenomic clusters. Dots indicate the metagenomes samples. Distances were calculated based on the Bray-Curtis Method. NMDS stress value = 0.15. [file Image4.TIF]
