# Supplementary material for: Ecogenomics and Taxonomy of Cyanobacteria Phylum
Source: Front Microbiol. 2017 Nov 14;8:2132. doi: 10.3389/fmicb.2017.02132 (PMC5694629; doi:10.3389/fmicb.2017.02132)
Supplement: Data Sheet 1 — Formal description of new genera and species. [file DataSheet1.DOCX]

# **Supplementary Material**

Ecogenomics and taxonomy of Cyanobacteria phylum

Juline M. Walter^1,2^, Felipe H. Coutinho^1,2^, Bas E. Dutilh^2,4^, Jean Swings^5^, Fabiano L. Thompson^1,3*^, and Cristiane C. Thompson^1*^

***** Corresponding Authors: fabianothompson1@gmail.com (FLT); thompsoncristiane@gmail.com (CCT)

# Formal description of new genera and species

**Description of *Adonisia* gen. nov.**

*Adonisia* (A.don.i.sia. Referring to Adonis, a divine figure in Greek mythology of oriental origin; -*ousia*, suffix meaning to be).

Description of *Adonisia splendidus* (formerly known as *Leptolyngbya* sp.) (splen.di.dus. L. adj. *splendidus*, brilliant): This species is characterized by β-carboxysome. Type strain is PCC 7375, isolated from marine habitat in Woods Hole, Massachusetts, USA. The genome of this strain contains 9,42 Mbp (GC = 47,62%) harboring 8,366 coding DNA sequences.

**Description of *Allocoleopsis* gen. nov.**

*Allocoleopsis* (allo.cole.opsis. Gr. adj. *allos*, other; N.L. n. *coleus* related to sheath; Gr. neut. n. *nema* thread, filament; Gr. suffix –*opsis*, looking like. *Allocoleopsis,* another cell with sheath).

Description of *Allocoleopsis franciscanus* (formerly known as *Microcoleus* sp.) (fran.cis.ca.nus. *franciscanus*, referring to the isolation area, San Francisco): This species is characterized by β-carboxysome. Type strain is PCC 7113, isolated from soil habitat in San Francisco, California, USA. The genome of this strain contains 7,47 Mbp (GC = 46,21%) harboring 6,734 coding DNA sequences.

**Description of *Arhinema* gen. nov.**

*Arhinema* (Arh.i.nema. Gr. adj. *arkhi*, the former, early; Gr. n. *nema*, thread; M. L. fem. n. *Arhinema*, another filament).

*Arhinema longislandicus* (formerly known as *Leptolyngbya* sp.) (long.is.lan.di.cus. referring to the habitat collected, Long Island, NY, USA): This species is characterized by β-carboxysome. Type strain is PCC 7104, isolated from marine habitat rockshore, Montauk Point, Long Island, NY, USA. The genome of this strain contains 6,89 Mbp (GC = 57,69%) harboring 6,414 coding DNA sequences.

**Description of *Calotaxis* gen. nov.**

*Calotaxis* (Ca.lo.ta.xis. Gr. *calo*, beautiful; Gr. *taxis*, arrangement; *Calotaxis*, beautiful arrangement of the filament).

Description of *Calotaxis gracile* (formerly known as *Pseudoanabaena* sp.) (gra.ci.le, L. neut. adj. *gracile*, slender): This species is characterized by β-carboxysome. Type strain is PCC 7367, isolated from marine habitat in intertidal zone, Mexico. The genome of this strain contains 4.55 Mbp (GC = 46.31%) harboring 3,960 coding DNA sequences.

**Description of *Coccusdissimilis* gen. nov.**

*Coccusdissimilis* (Co.ccus.di.ssi.mi.lis. Gr. n. *kokkos*, grain or kernel; L. adj. *dissimilis*, different; *Coccusdissimilis*, a different coccos).

Description of *Coccusdissimilis mexicanus* comb. nov. (formerly known as *Synechococcus mexicanus*) (coccusdissimulis. Gr. n. *kokkos*, grain or kernel; L. adj. *dissimilis*, different; *Coccusdissimilis*, a different coccos): This species is characterized by β-carboxysome. Type strain is PCC 7335, isolated from marine habitat in Snail shell, intertidal zone, Puerto Penasco, Mexico. The genome of this strain contains 5.97 Mbp (GC = 48.20%) harboring 5,702 coding DNA sequences.

**Description of *Cyclospexia* gen. nov.**

*Cyclospexia* (Cy.clos.pex.ia. Gr. n. kyklos, cycle; Gr. *p*e*xia* (adj. -*pectic*), fixation; n. *Cyclospexia,* involved in N_2_ fixation cycle).

*Cyclospexia valenium* (formerly known as cyanobacterium) (val.e.nium. L. *valens*, strong; L. suff. -*icus* -*a* -*um*, suffix used with the sense of pertaining to; *valenium* referring to an organism restricted to few types of environmental conditions, such as extremophilic habitat): This species is characterized by β-carboxysome. Type strain is ESFC-1, isolated from marine habitat in extremophilic mat communities, Elkhorn Slough estuary, CA, USA. The genome of this strain contains 5.62 Mbp (GC = 46.51%) harboring 4,857 coding DNA sequences.

**Description of *Doliumcoccus* gen. nov.**

*Doliumcoccus* (Do.li.um.coc.cus. L. *dolium*, barrel; N.L. masc. n. *coccus* [from Gr. Masc. n. *kokkos*, grain, seed, kernel]; N.L masc. n. *Doliumcoccus*, referring to a circular cell shape).

Description of *Doliumcoccus* *switzii* (formerly known as *Synechocystis* sp.) (swi.t.zii. *switzii* referring to the local of origin, Switzerland): This species is characterized by β-carboxysome. Type strain is PCC 7509, isolated from soil habitat in Switzerland. The genome of this strain contains 4,9 Mbp (GC = 41,67%) harboring 4,859 coding DNA sequences.

**Description of *Enugrolinea* gen. nov.**

*Enugrolinea* (En.ugro.lin.ea. Gr. adj. *enugros* or *enhygro*, growing or living in wet habitats, in the water, aquatic; L. fem. n. *linea*, line; N. L. fem. n. *Enugrolinea*, filament of wet habitat, that means an aquatic strain).

Description of *Enugrolinea bermudensis* (formerly known as *Leptolyngbya* sp.) (ber.mu.den′sis. N.L. fem. adj. *bermudensis* from the Bermuda Islands, the geographical origin of the strain): This species is characterized by β-carboxysome. Type strain is PCC 7376, isolated from marine habitat in Limestone, Crystal Cave, Bermuda. The genome of this strain contains 5.12 Mbp (GC = 43.87%) harboring 4,601 coding DNA sequences.

Description of *Enugrolinea euryhalinus* comb. nov. (formerly known as *Synechococcus euryhalinus*) [c.f. *S. euryhalinus*]: This species is characterized by β-carboxysome. Type strain is PCC 7002. The genome of this strain contains 3.41 Mbp (GC = 49.16%) harboring 3,121 coding DNA sequences.

**Description of *Brevicoccus* gen. nov.**

*Brevicoccus* (Brev.i.co.ccus. L. *brevis*, short, small; N.L. masc. n. *coccus* [from Gr. Masc. n. *kokkos*, grain, seed, kernel]; N.L masc. n. *Brevicoccus*, a small coccus).

Description of *Brevicoccus berkleyi* comb. nov. (formerly known as *Synechococcus berkleyi*) [c.f. *S. berkleyi*]: This species is characterized by β-carboxysome. Type strain is PCC 7336, isolated from marine habitat in Sea Water Tank, Berkeley University, CA, USA. The genome of this strain contains 5.07 Mbp (GC = 53.70%) harboring 5,093 coding DNA sequences.

**Description of *Eurycolium* gen. nov.**

*Eurycolium* (Eur.y.co.lium. Gr. adj. *eury*, wide, broad; L. *cole*, inhabit; Gr. –*ium*, quality or relationship, *Eurycolium,* referring to the spread inhabiting trait in marine habitats).

Description of *Eurycolium pastoris* (formerly known as *Prochlorococcus pastoris*) [c.f. *P. pastoris*]: This species is characterized by α-carboxysome. Type strain is CCMP 1986 (or MED4), isolated from marine habitat. The genome of this strain contains 1,65 Mbp (GC = 30,8%) harboring 1,777 coding DNA sequences.

Description of *Eurycolium tetisii* (formerly known as *Prochlorococcus tetisii*) [c.f. *P. tetisii*]: This species is characterized by α-carboxysome. Type strain is MIT 9515, isolated from marine habitat. The genome of this strain contains 1,7 Mbp (GC = 30,79%) harboring 1,784 coding DNA sequences.

Description of *Eurycolium neptunius* (formerly known as *Prochlorococcus neptunius*) [c.f. *P. neptunius*]: This species is characterized by α-carboxysome. Type strain is MIT 9312, isolated from marine habitat. The genome of this strain contains 1,7 Mbp (GC = 31,21%) harboring 1,815 coding DNA sequences.

Description of *Eurycolium ponticus* (formerly known as *Prochlorococcus ponticus*) [c.f. *P. ponticus*]: This species is characterized by α-carboxysome. Type strain is MIT 9301, isolated from marine habitat. The genome of this strain contains 1,64 Mbp (GC = 31,34%) harboring 1,774 coding DNA sequences.

Description of *Eurycolium nereus* (formerly known as *Prochlorococcus nereus*) [c.f. *P. nereus*]: This species is characterized by α-carboxysome. Type strain is MIT 9202, isolated from marine habitat. The genome of this strain contains 1,69 Mbp (GC = 31,1%) harboring 1,795 coding DNA sequences.

Description of *Eurycolium chisholmii* (formerly known as *Prochlorococcus chisholmii*) [c.f. *P. chisholmii*]: This species is characterized by α-carboxysome. Type strain is AS9601, isolated from marine habitat. The genome of this strain contains 1,66 Mbp (GC = 31,32%) harboring 1,769 coding DNA sequences.

**Description of *Euryforis* gen. nov.**

*Euryforis* (Eur.y.fo.rys. Gr. masc. n. *eury*, broad; N.L. masc. n. *foris*, outside; N.L. masc. n. *Euryforis,* a widespread genus).

Description of *Euryforis eilemai* (formerly known as *Leptolyngbya* sp.) (ei.le.mai. N.L. gen. n. *eilemai*, referring to the presence of sheath): This species is characterized by β-carboxysome. Type strain is PCC 6406, isolated from freshwater habitat in California, USA. The genome of this strain contains 5,77 Mbp (GC = 55,18%) harboring 5,156 coding DNA sequences.

**Description of *Inmanicoccus* gen. nov.**

*Inmanicoccus* (Inm.a.ni.co.ccus. L. *inmani*, huge, vast, immense, referring to an abundant and broad organism).

Description of *Inmanicoccus mediterranei* comb. nov. (formerly known as *Parasynechococcus* *mediterranei*) (me.di.ter.ra.nei L. masc. adj., referring to the increased abundance of this organism at the Mediterranean province): This species is characterized by α-carboxysome. Type strain is RCC 307, isolated from marine habitat in Mediterranean Sea (15 m depth). The genome of this strain contains 2,22 Mbp (GC = 60,8%) harboring 2,348 coding DNA sequences.

***Description of Leptococcus gen. nov.***

*Leptococcus* (Lept.o.co.ccus. Gr. adj. *l*eptos, delicate or thin; N.L. masc. n. coccus [from Gr. Masc. n. *kokkos*, grain, seed, kernel]; N.L masc. n. *Leptococcus*, a delicate coccos).

Description of *Leptococcus springii* com. nov. (formerly known as *Synechococcus springii*) [c.f. *S. springii*]: This species is characterized by β-carboxysome. Type strain is JA23Ba213, isolated from freshwater habitat in Octopus Spring, Yellowstone Park, USA. The genome of this strain contains 3.05 Mbp (GC = 58.50%) harboring 3,064 coding DNA sequences.

Description of *Leptococcus yellowstonii* comb. nov. (formerly known as *Synechococcus yellowstonii*) [c.f. *S. yellowstonii*]: This species is characterized by β-carboxysome. Type strain is JA33Ab, isolated from freshwater habitat in Octopus Spring, Yellowstone Park, USA. The genome of this strain contains 2.93 Mbp (GC = 60.20%) harboring 3,036 coding DNA sequences.

**Description of *Leptolatis* gen. nov.**

*Leptolatis* (Lep.to.la.tis. Gr. adj. *l*eptos, delicate or thin; L. n. *latus*, broad, wide; *Leptolatis,* referring to a wide genus).

Description of *Leptolatis* *gracile* (formerly known as *Pseudanabaena* sp.) (grac.i.le. L. *gracile*, referring to gracefully, slender): This species is characterized by β-carboxysome. Type strain is PCC 7367, isolated from marine habitat. The genome of this strain contains 4,55 Mbp (GC = 46,31%) harboring 3,960 coding DNA sequences.

**Description of *Leptovivax* gen. nov.**

*Leptovivax* (Lept.o.vi.vax. Gr. adj. *l*eptos, delicate or thin; L. adj. *v*ivax, long-lived, tenacious of life; *Leptovivax*, delicate long-lived genus).

Description of *Leptovivax bogii* (formerly known as *Synechococcus bogii*) [c.f. *S. bogii*]: This species is characterized by β-carboxysome. Type strain is PCC 7502, isolated from a sphagnum bog habitat. The genome of this strain contains 3.58 Mbp (GC = 40.60%) harboring 3,703 coding DNA sequences.

**Description of *Magnicoccus* gen. nov.**

*Magnicoccus* (Mag.ni.co.ccus. L. *magni*, vast, extensive; N.L. masc. n. *coccus* [from Gr. Masc. n. *kokkos*, grain, seed, kernel]; N.L masc. n. *Magnicoccus,* referring to an abundant and broad organism).

Description of *Magnicoccus* *indicus* comb. nov. (formerly known as *Parasynechococcus* *indicus*) (in.di.cus NL., referring to the increased abundance of this organism at the Indian Ocean provinces): This species is characterized by α-carboxysome. Type strain is CB 0205. The genome of this strain contains 2,43 Mbp (GC = 63%) harboring 2,473 coding DNA sequences.

Description of *Magnicoccus* *sudiatlanticus* comb. nov. (formerly known as *Parasynechococcus* *sudiatlanticus*) (su.di.a.tlan.ti.cus NL., referring to the increased abundance of this organism at the South Atlantic province): This species is characterized by α-carboxysome. Type strain is CB 0101, isolated from marine habitat in Chesapeake Bay, Baltimore, Maryland, USA. The genome of this strain contains 2,69 Mbp (GC = 64,2%) harboring 2,757 coding DNA sequences.

**Description of *Thaumococcus* gen. nov.**

*Thaumococcus* (Thau.mo.co.ccus. Referring to Thaumas, Greek god of the wonders of the sea; N.L. masc. n. *coccus* [from Gr. Masc. n. *kokkos*, grain, seed, kernel]; N.L masc. n. *Thaumococcus*, referring to a coccus living in the sea).

Description of *Thaumococcus swingsii* comb. nov. (formerly known as *Prochlorococcus swingsii*) [c.f. *P. swingsii*]: This species is characterized by α-carboxysome. Type strain is MIT 9313, isolated from a marine habitat in Gulf Stream (135 m depth). The genome of this strain contains 2,41 Mbp (GC = 50,74%) harboring 2,339 coding DNA sequences.

**Description of *Paraleptovivax* gen. nov.**

*Paraleptovivax* (Pa.ra.lep.to.vi.vax. *para*, to bring forth, to bear, alongside; Gr. adj. *l*eptos, delicate or thin; L. adj. *v*ivax, long-lived, tenacious of life; *Leptovivax*, delicate long-lived).

Description of *Paraleptovivax allomegium* (formerly known as *Pseudoanabaena* sp.) (all.o.me.gium. Gr. adj. *allos*, other; Gr. nom. neut. adj. m*ega*, big; Gr. –*ium*, quality or relationship): This species is characterized by β-carboxysome. Type strain is PCC 6802, isolated from freshwater habitat in California, USA. The genome of this strain contains 5.62 Mbp (GC = 47.83%) harboring 5,363 coding DNA sequences.

**Description of *Paraspirulina* gen. nov.**

*Paraspirulina* (Par.as.pi.ru.li.na. Gr. adj. *para*, to bring forth, to bear, alongside; *Spirulina*, other genus of cyanobacteria [cf. genus *Spirulina*]; M.L. fem. n. *Paraspirulina*).

Description of *Paraspirulina subsalsa* comb. nov. (formerly known as *Spirulina subsalsa*) [c.f. *S. subsalsa*]: This species is characterized by β-carboxysome. Type strain is PCC 9445. The genome of this strain contains 5,32 Mbp (GC = 47,39%) harboring 4,580 coding DNA sequences.

**Description of *Prolificoccus* gen. nov.**

*Prolificoccus* (Pro.li.fi.co.ccus. L. *prolificus*, productive, abundant, numerous; *Prolificoccus*, referring to an abundant coccus).

Description of *Prolificoccus proteus* comb. nov. (formerly known as *Prochlorococcus proteus*) [c.f. *P. proteus*]: This species is characterized by α-carboxysome. Type strain is NATL2A, isolated from a marine habitat in Northern Atlantic (10 m depth). The genome of this strain contains 1,84 Mbp (GC = 35,12%) harboring 1,930 coding DNA sequences.

**Description of *Pseudogeitlerinema* gen. nov.**

*Pseudogeitlerinema* (Pseudo.gei.tle.ri.ne.ma. Gr. adj. *pseud*, false*; Geitlerinema*, another cyanobacteria genus [cf. genus *Geitlerinema*]; M. L. fem. n. *Pseudogeitlerinema*).

Description of *Pseudogeitlerinema shalloid* (formerly known as *Geitlerinema* sp.) (shal.loid. Gr. *-oid*, resembling, having the appearance of; *shalloid* referring to the appearance of very shallow adjacent cells): This species is characterized by β-carboxysome. Type strain is PCC 7407. The genome of this strain contains 4,68 Mbp (GC = 58,46%) harboring 3,727 coding DNA sequences.

**Description of *Pseudosynechococcus* gen. nov.**

*Pseudosynechococcus* (Pseudo.sy.ne.cho.co.ccus. Gr. adj. *pseud*, false*; Synechococcus*, another cyanobacteria genus [cf. genus *Synechococcus*]).

Description of *Pseudosynechococcus benguelii* comb. nov. (formerly known as *Parasynechococcus* *benguelii*) (ben.gue.lii. NL., referring to the increased abundance of this organism at the Benguela current coastal province): This species is characterized by α-carboxysome. Type strain is CC 9311, isolated from marine habitat in California current, Pacific, coastal, 95 m. The genome of this strain contains 2,61 Mbp (GC = 52,4%) harboring 2,627 coding DNA sequences.

Description of *Pseudosynechococcus pacificus* comb. nov. (formerly known as *Parasynechococcus* *pacificus*) (pa.ci.fi.cus L. masc. adj. Referring to the increased abundance of this organism at the Pacific Ocean provinces): This species is characterized by α-carboxysome. Type strain is WH 7803, isolated from marine habitat in Sargasso Sea (25 m depth). The genome of this strain contains 2,37 Mbp (GC = 60,2%) harboring 2,439 coding DNA sequences.

Description of *Pseudosynechococcus subtropicalis* comb. nov. (formerly known as *Parasynechococcus* *subtropicalis*) (sub.tro.pi.ca.lis NL. Referring to the increased abundance of this organism at Subtropical provinces): This species is characterized by α-carboxysome. Type strain is WH 7805, isolated from marine habitat in Sargasso Sea. The genome of this strain contains 2,63 Mbp (GC = 57,6%) harboring 2,595 coding DNA sequences.

Description of *Pseudosynechococcus sudipacificus* comb. nov. (formerly known as *Parasynechococcus* *sudipacificus*) (su.di.pa.ci.fi.cus NL. Referring to the increased abundance of this organism at South Pacific provinces): This species is characterized by α-carboxysome. Type strain is WH 8016, isolated from marine habitat in Woods Hole, MA, USA. The genome of this strain contains 2,69 Mbp (GC = 54,1%) harboring 2,990 coding DNA sequences.

**Description of *Regnicoccus* gen. nov.**

*Regnicoccus* (Regn.i.co.ccus. L. *regni*, powerful, royal power, control, referring to an abundant organism.

Description of *Regnicoccus* *antarcticus* comb. nov. (formerly known as *Parasynechococcus* *antarcticus*) (ant.arc.ti.cus. L. masc. adj., referring to the increased abundance of this organism at the Antarctic province): This species is characterized by α-carboxysome. Type strain is WH 5701, isolated from marine habitat in Long Island Sound, Connecticut, USA. The genome of this strain contains 3,28 Mbp (GC = 65,4%) harboring 2,917 coding DNA sequences.

**Description of *Rheamaris* gen. nov.**

*Rheamaris* (Rhea.mar’is. Referring to Rhea, the fertile Greek goddess, mother of some of the most well-known Greek gods and goddesses, yet she is often forgotten; L. gen. n. *maris*, of the sea, marine; *Rheamaris*, fertile goddess of seawater).

Description of *Rheamaris confervoides* comb. nov. (formerly known as *Lyngbya confervoides*) [cf. species *L. confervoides*]: This species is characterized by β-carboxysome. Type strain is BDU (or BDU141951), isolated from a marine habitat in India. The genome of this strain contains 8,79 Mbp (GC = 55,63%) harboring 8,370 coding DNA sequences.

**Description of *Rotundosa* gen. nov.**

*Rotundosa* (Ro.tund.osa. L. *rotundus*, circular; Rotundosa, referring to their round shape).

Description of *Rotundosa thermolimnetic* (formerly known as *Gloeocapsa* sp.) (ther.mo.lim.ne.tic. Gr. adj. *thermos*, hot; N.L. fem. adj. *limnetica* (from Gr. n. *limnê*), lake; N.L. gen. n. *thermolimnetic*, referring to the habitat, freshwater thermal springs): This species is characterized by β-carboxysome. Type strain is PCC 7428, isolated from freshwater habitat in moderate hot spring. The genome of this strain contains 5,43 Mbp (GC = 43,27%) harboring 5,254 coding DNA sequences.

**Description of *Somacatellium* gen. nov.**

*Somacatellium* (Som.a.ca.te.llium. Gr. n. *soma* (*somatiko*), body; L. fem. n. *catella*, a small chain; *Somacatellium* referring to a small chain filamentous body).

*Somacatellium hydroxylic* (formerly known as *Oscillatoria* sp.) (hy.drox.y.lic. Gr. -*ic (ikos)*, relating to or having some characteristic of; *hydroxylic* referring to the hydroxyl-proline and hydroxyl aspartic acid compounds the new cinnamycin variant found in this strain): This species is characterized by β-carboxysome. Type strain is PCC 10802. The genome of this strain contains 8,59 Mbp (GC = 54,1%) harboring 7,012 coding DNA sequences.

**Description of *Stenotopis* gen. nov.**

*Stenotopis* (Ste.no.to.pis. Gr. adj. *stenos*, narrow; Gr. n. *topos*, place; *Stenotopis* referring a living within a narrow range of places).

Description of *Stenotopis californii* comb. nov. (formerly known as *Synechococcus californii*) (Ste.no.to.pis. Gr. adj. *stenos*, narrow; Gr. n. *topos*, place; *Stenotopis* referring a living within a narrow range of places): This species is characterized by β-carboxysome. Type strain is PCC 6312, isolated from freshwater habitat in California, USA. The genome of this strain contains 3.72 Mbp (GC = 48.49%) harboring 3,795 coding DNA sequences.

**Description of *Tapinonema* gen. nov.**

*Tapinonema (ta.pi.no.nema. Gr. tapino small*, modest, weak; Gr. n. *nema*, thread; *Tapinonema*, small filament).

Description of *Tapinonema colecalium* (formerly known as cyanobacterium) (col.e.ca.lium. L. *cole*, inhabit; Gr. *calo*, beautiful; –*ium*, quality or relationship; *colecalium*, beautiful inhabitant): This species is characterized by β-carboxysome. Type strain is JSC-12, isolated from freshwater habitat. The genome of this strain contains 5,52 Mbp (GC = 47,49%) harboring 5,024 coding DNA sequences.

**Description of *Toxinema* gen. nov.**

*Toxinema* (tox.i.nema. N.L. n. *toxi* toxins related to the strains; Gr. neut. n. *nema* thread, filament; N.L. neut. n. *Toxinema* a filament with release toxins).

Description of *Toxinema oscillati* (formerly known as *Oscillatoria* sp.) (os.ci.lla.ti. Gr. o*scillare*, to swing, *Oscillatoria* is other genus of cyanobacteria [cf. genus *Oscillatoria*]): This species is characterized by β-carboxysome. Type strain is PCC 6407, isolated from freshwater habitat. The genome of this strain contains 6,89 Mbp (GC = 43,43%) harboring 5,693 coding DNA sequences.

# Formal description of new species (within known genera)

***Anabaena genus***

Description of *Anabaena mossii* (formerly known as *Anabaena* sp.) (mos.si. *mossi*, referring to the isolation area, intertidal zone, Moss Beach, CA, USA): This species is characterized by β-carboxysome. Type strain is PCC 7108, isolated from marine habitat in intertidal zone, Moss Beach, CA, USA. The genome of this strain contains 5,9 Mbp (GC = 38,78%) harboring 5,169 coding DNA sequences.

***Arthrospira genus***

Description of *Arthrospira nitrilium* (formerly known as *Arthrospira* sp.) (ni.tri.lium. N.L. gen. n. *nitrilium*, referring to the capacity to utilize nitriles (R-C≡N) as the sole source of nitrogen): this species is characterized by β-carboxysome and the phycobilisome pigmentation of this strain has not been characterized. Type strain is PCC 8005, isolated from a freshwater habitat in alkaline salt lakes. The genome of this strain contains 6,27 Mbp (GC = 44,7%) harboring 5,171 coding DNA sequences.

Description of *Arthrospira sesilensis* (formerly known as *Arthrospira platensis*) (ses.i.len.sis. N.L. gen. n. *sesilensis*, referring to the lack of gliding): this species is characterized by β-carboxysome and the phycobilisome pigmentation of this strain has not been characterized. Type strain is PCC 9438 (or C1), isolated from a freshwater habitat in alkaline salt lakes. The genome of this strain contains 6 Mbp (GC = 44,69%) harboring 4,852 coding DNA sequences.

***Dactylococcopsis genus***

Description of *Dactylococcopsis halotolerans* (formerly known as *Halothece* sp.) (ha.lo.to.le.rans*.* Gr. n. *hals*, *halos*, salt, sea; L. pres. part. *tolerans*, tolerating; M. L. part. adj. Halotolerans salt-tolerating): This species is characterized by β-carboxysome. Type strain is PCC 7418, isolated from freshwater habitat in Solar Lake, Israel. The genome of this strain contains 4,18 Mbp (GC = 42,92%) harboring 3,663 coding DNA sequences.

***Fischerella* genus**

Description of *Fischerella* *welwii* (formerly known as *Fischerella* sp.) (wel.wii. *welwii*, referring to the presence of welwitindolinone gene cluster, a hapalindole-type family of natural products): This species is characterized by β-carboxysome. Type strain is PCC 9431. The genome of this strain contains 7,16 Mbp (GC = 40,19%) harboring 6,104 coding DNA sequences.

Description of *Fischerella* *hapalii* (formerly known as *Fischerella* sp.) (hap.a.lii. *hapalii*, referring to the presence of hapalindole gene cluster, a hapalindole-type family of natural products): This species is characterized by β-carboxysome. Type strain is PCC 9339. The genome of this strain contains 8 Mbp (GC = 40,16%) harboring 6,720 coding DNA sequences.

Description of *Fischerella* *sesquitii* (formerly known as *Fischerella* sp.) (ses.qui.tii. *sesquitii*, referring to the presence of sesquiterpene gene cluster, encoding three proteins): This species is characterized by β-carboxysome. Type strain is JSC-11. The genome of this strain contains 5,38 Mbp (GC = 41,05%) harboring 4,627 coding DNA sequences.

Description of *Fischerella* *peptidasii* (formerly known as *Fischerella* sp.) (pep.ti.da.sii. *peptidasii*, referring to the presence of genes encoding peptidases, such as M16, characteristic of the group II bacteriocin gene clusters): This species is characterized by β-carboxysome. Type strain is PCC 9605, isolated from soil (limestone) habitat in Jerucham, Har Rahama, Israel. The genome of this strain contains 8,08 Mbp (GC = 42,61%) harboring 7,060 coding DNA sequences.

***Geitlerinema genus***

Description of *Geitlerinema catellasis* (formerly known as *Geitlerinema* sp.) (cat.e.lla.sis. L. fem. n. *catella*, referring to the filamentous small chain): This species is characterized by α-carboxysome. Type strain is PCC 7105, isolated in USA. The genome of this strain contains 6,15 Mbp (GC = 51,59%) harboring 4,735 coding DNA sequences.

***Geminocystis genus***

Description of *Geminocystis stanieri* comb. nov. (formerly known as *Cyanobacterium stanieri*) [c.f. *C. stanieri*]: This species is characterized by β-carboxysome. Type strain is PCC 7202, isolated from freshwater habitat in thermal spring, alkaline pod. The genome of this strain contains 3,16 Mbp (GC = 38,66%) harboring 2,886 coding DNA sequences.

***Lyngbya genus***

Description of *Lyngbya limosa* (formerly known as *Lyngbya* sp.) (li.mo.sa. *limosa*, referring to the former nomenclature [c.f. *Oscillatoria limosa*]): This species is characterized by β-carboxysome. Type strain is PCC 8106. The genome of this strain contains 7,03Mbp (GC = 41,11%) harboring 5,854 coding DNA sequences.

***Microcoleus* genus**

Description of *Microcoleus nigroviridis* comb. nov. (formerly known as *Oscillatoria nigroviridis*) (nig.ro.vi.ri.dis. Gr. adj. *nigro*, distinctus; Gr. adj. *viridis*, campus, a green field; *Oscillatoria* is other genus of cyanobacteria [cf. genus *Oscillatoria*]): This species is characterized by β-carboxysome. Type strain is PCC7112, isolated from a soil habitat in USA. The genome of this strain contains 7,47 Mbp (GC = 45,87%) harboring 6,925 coding DNA sequences.

***Nostoc* genus**

Description of *Nostoc amparaii* (formerly known as *Nostoc* sp.) [c.f. genus *Nostoc*] (am.pa.raii. amparaii, referring to the isolation area, Amparai District, Sri Lanka): This species is characterized by β-carboxysome. Type strain is PCC 7524, isolated from freshwater habitat in hot spring, Amparai District, Maha Oya, Sri Lanka. The genome of this strain contains 7,61 Mbp (GC = 42,2%) harboring 5,326 coding DNA sequences.

Description of *Nostoc reyesii* (formerly known as *Nostoc* sp.) [c.f. genus *Nostoc*] (rey.es.si. *reyessi*, referring to the isolation area, Point Reyes Peninsula, CA, USA): This species is characterized by β-carboxysome. Type strain is PCC 7107, isolated from freshwater habitat in Point Reyes Peninsula, CA, USA. The genome of this strain contains 6,32 Mbp (GC = 40,36%) harboring 5,200 coding DNA sequences.

***Planktothrix genus***

Description of *Planktothrix stereotis* comb. nov. (formerly known as *Planktothrix* *agardhii*) (ster.eo.tis. Gr. *stereos*, solid; *stereotis*, referring to a solid and straight filament): This species is characterized by β-carboxysome. Type strain is NIVA-CYA 126, isolated from freshwater habitat. The genome of this strain contains 5,04 Mbp (GC = 39,57%) harboring 4,188 coding DNA sequences.

***Pleurocapsa* genus**

Description of *Pleurocapsa penascus* (formerly known as *Pleurocapsa* sp.) (pe.nas.cus. *penascus*, referring to the isolation area, Puerto Penasco): This species is characterized by β-carboxysome. Type strain is PCC 7319, isolated from marine habitat in Arizona Station, Gulf of California, Puerto Penasco, Mexico. The genome of this strain contains 7,38 Mbp (GC = 38,74%) harboring 4,516 coding DNA sequences.

***Xenococcus genus***

Description of *Xenococcus lajollai* (formerly known as *Xenococcus* sp.) (la.jo.llai. *lajollai*, referring to the isolation area, La Jolla Aquarium, CA, USA): This species is characterized by β-carboxysome. Type strain is PCC 7305, isolated from marine habitat in Aquarium, La Jolla, CA, USA. The genome of this strain contains 5,92 Mbp (GC = 39,68%) harboring 4,992 coding DNA sequences.

# Abbreviations

adj. adjective; Gr. Greek; L. Latin; n. noun; neut. neutro; c.f. confer, conferre; v. verb; CDS protein-coding sequence; NA "Not Available" or "Not Applicable" or "Not Announced”.
